# Factors affecting the mixed-layer concentrations of singlet oxygen in sunlit lakes†

**DOI:** 10.1039/d1em00062d

**Published:** 2021-06-23

**Authors:** Sarah B. Partanen, Jennifer N. Apell, Jianming Lin, Kristopher McNeill

**Affiliations:** Institute of Biogeochemistry and Pollutant Dynamics (IBP), Department of Environmental Systems Science, ETH Zurich 8092 Zurich Switzerland japell@nyu.edu kristopher.mcneill@env.ethz.ch; Department of Civil and Urban Engineering, New York University Tandon School of Engineering 6 MetroTech Center Brooklyn NY 11201 USA; Firmenich Incorporated P.O. Box 5880 Princeton New Jersey 08543 USA

## Abstract

The steady-state concentration of singlet oxygen within a lake ([^1^O_2_]_SS_) is an important parameter that can affect the environmental half-life of pollutants and environmental fate modelling. However, values of [^1^O_2_]_SS_ are often determined for the near-surface of a lake, and these values typically do not represent the average over the epilimnia of lakes. In this work, the environmental and physical factors that have the largest impact on [^1^O_2_]_SS_ within lake epilimnia were identified. It was found that the depth of the epilimnion has the largest impact on depth-averaged [^1^O_2_]_SS_, with a factor of 8.8 decrease in [^1^O_2_]_SS_ when epilimnion depth increases from 2 m to 20 m. The next most important factors are the wavelength-dependent singlet oxygen quantum yield relationship and the latitude of the lake, causing variations in [^1^O_2_]_SS_ by factors of 3.2 and 2.5 respectively, over ranges of representative values. For a set of representative parameters, the depth-averaged value of [^1^O_2_]_SS_ within an average epilimnion depth of 9.0 m was found to be 5.8 × 10^−16^ M and the near-surface value of [^1^O_2_]_SS_ was found to be 1.9 × 10^−14^ M. We recommend a range of 6 × 10^−17^ to 5 × 10^−15^ M as being more representative of [^1^O_2_]_SS_ values within the epilimnia of lakes globally and potentially more useful for estimating pollutant lifetimes than those calculated using [^1^O_2_]_SS_ values that correspond to near-surface, summer midday values. This work advances our understanding of [^1^O_2_]_SS_ inter-lake variability in the environment, and provides estimates of [^1^O_2_]_SS_ for practitioners and researchers to assess environmental half-lives of pollutants due to reaction with singlet oxygen.

## Abstract 2

[^1^O_2_]_SS_ within lake epilimnia are highly affected by epilimnion depth and are barely affected by DOC concentration within the lake.

## Graphical abstract

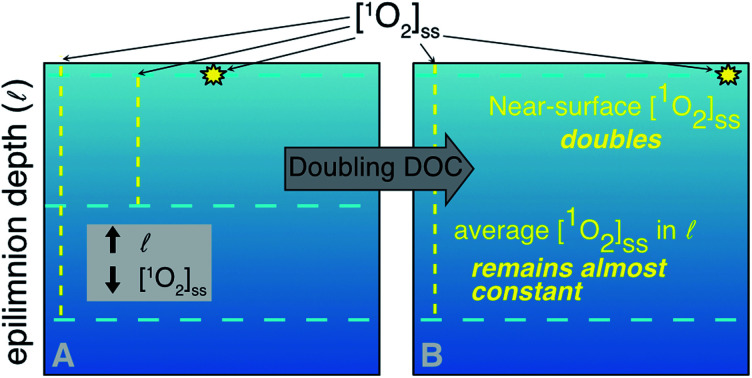

**Environmental significance**

Reaction with singlet oxygen has been shown to be an important degradation pathway for several aqueous organic pollutants, and the environmental half-life for this reaction pathway strongly depends on the steady-state concentration of singlet oxygen ([^1^O_2_]_SS_). While [^1^O_2_]_SS_ have been measured for many surface waters, reported values are often representative of near-surface conditions, and are not representative of [^1^O_2_]_SS_ averaged over the epilimnia of lakes. This work shows that average [^1^O_2_]_SS_ in lake epilimnia can be up to two orders of magnitude lower than what is typically measured at the near-surface. The epilimnion depth was found to have the greatest impact on the depth-averaged [^1^O_2_]_SS_, whereas the dissolved organic carbon concentration was found to have a limited impact. Using depth-averaged [^1^O_2_]_SS_ values allows for more accurate predictions of pollutant half-lives within lakes due to reaction with singlet oxygen.

## Introduction

Photochemical processes in surface waters such as lakes and rivers are important pathways for the degradation of some organic contaminants.^1–4^ While some photodegradable compounds react following the direct absorption of photons, others, such as the pesticide fludioxonil^5^ and the pharmaceutical cimetidine,^6^ are mainly attenuated *via* indirect photochemical processes. Indirect photochemistry is distinguished by the formation of photochemically produced reactive intermediates (PPRI), often through absorption of light by the chromophoric fraction of dissolved organic matter (CDOM).^7–9^ One PPRI of importance is singlet oxygen (^1^O_2_), a reactive oxygen species that selectively reacts with functional groups such as cyclic dienes, heterocycles, and reduced sulfur-containing compounds.^6,10,11^

While the processes by which ^1^O_2_ is formed and quenched in surface waters are relatively well understood,^11–13^^1^O_2_ steady-state concentrations ([^1^O_2_]_SS_) in the natural environment are less well defined. This is partly due to the variability caused by environmental conditions such as latitude, epilimnion depth, and the dissolved organic carbon (DOC) concentration in a water body. There is also added uncertainty due to limited experimental data characterizing parameters such as the wavelength-dependent ^1^O_2_ quantum yields (*Φ*_Δ,*λ*_), the fraction of total absorbance that is attributable to CDOM (*f*_a,CDOM_), and the fraction of light that is backscattered (*f*_backscatter_) out of the water column. Understanding how these factors affect [^1^O_2_]_SS_ would help constrain possible values of [^1^O_2_]_SS_, which in turn can be used to more accurately quantify the role of ^1^O_2_ in pollutant degradation and biogeochemical processes in the natural environment.

Typically, ^1^O_2_ in natural waters is investigated by taking a surface water sample from the field and conducting laboratory measurements to determine the quantum yield and the [^1^O_2_]_SS_.^13–16^ Singlet oxygen quantum yield measurements require accurate characterization of the incident light, making these results independent of the light source when performed correctly.^17^ In contrast, measurements of [^1^O_2_]_SS_ do not require light source characterization, so values reported by different researchers or at different times may not be comparable. Additionally, laboratory measurement conditions are not generally representative of conditions in the environment. Specifically, laboratory light sources often do not replicate the solar spectrum, and the irradiance is usually substantially higher in these systems. In some cases, laboratory light spectra are designed to mimic solar noon or “sunny summer day” solar irradiance spectra,^18–20^ but even these spectra are not representative of the sunlight received by a water body over an entire day or year. Similarly, natural water samples examined in the laboratory tend to be more representative of surface conditions.^13,18,21–23^ Half-lives based on ideal conditions (summer, midday, near-surface) are overly rosy with respect to the importance of reaction with ^1^O_2_. A step toward more realistic values would be to instead use [^1^O_2_]_SS_ values that are averaged over the epilimnion (mixed layer) of a lake.

Modelling photochemical processes can generate more environmentally relevant results compared to measuring samples in the laboratory because a range of values representing diverse geographical locations and surface waters can be more easily simulated.^24,25^ However, current models have important limitations such as (1) using only a representative value instead of a range of values that capture uncertainty or variability for some input parameters, (2) focusing only on direct photolysis, or (3) having low ease of use.^24,26,27^ Additionally, a systematic sensitivity analysis of the parameters affecting ^1^O_2_ formation and quenching in natural surface waters has not been addressed.

In this work the variability of [^1^O_2_]_SS_ in a wide range of theoretical surface waters was investigated by examining the effect on [^1^O_2_]_SS_ due to epilimnion depth, incident irradiance, singlet oxygen quantum yield, dissolved organic carbon concentration, and fraction of absorbance attributable to CDOM. Which parameters introduce the most uncertainty and variability into predictions of [^1^O_2_]_SS_ has also been explored. Using the predicted [^1^O_2_]_SS_ values, a range of potential environmental half-lives of organic contaminants due to reaction with ^1^O_2_ was estimated. The [^1^O_2_]_SS_ values reported here are valuable for practitioners wishing to estimate the environmental half-lives of specific chemical compounds in diverse surface waters as well as for researchers interested in the environmental variability of singlet oxygen.

## Methods

### Governing equations for estimating [^1^O_2_]_SS_

The average [^1^O_2_]_SS_ within the epilimnion of a lake was estimated based on the following relationships:1
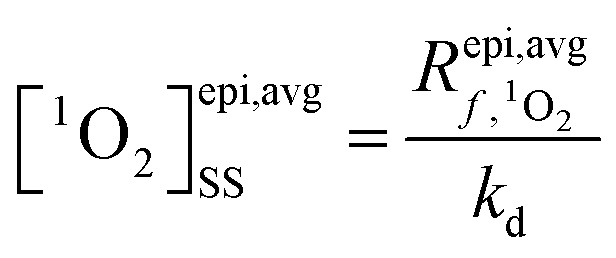
2
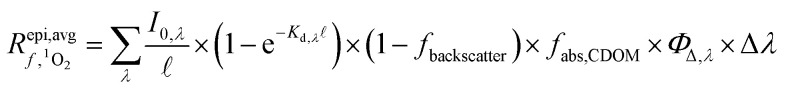
where 
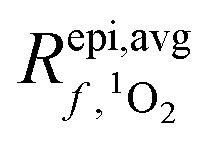
 is the average rate of formation of ^1^O_2_ throughout the epilimnion (M s^−1^), *k*_d_ is the deactivation rate constant for ^1^O_2_ in water (s^−1^), *I*_0,*λ*_ is the incident irradiance (mmol photons cm^−2^ s^−1^ nm^−1^), 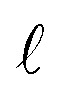 is the epilimnion depth (cm), *K*_d,*λ*_ is the diffuse attenuation coefficient (cm^−1^), *f*_backscatter_ is the fraction of light that is backscattered out of the water column, *f*_abs,CDOM_ is the fraction of the total absorbance that is attributable to absorbance by CDOM, *Φ*_Δ,*λ*_ is the wavelength-dependent singlet oxygen quantum yield, and Δ*λ* is the wavelength interval for the summation, here 1 nm. In this work we use [^1^O_2_]^epi,avg^_SS_ to refer to the singlet oxygen steady-state concentration averaged over the epilimnion depth of a lake, and [^1^O_2_]_SS_ to refer to singlet oxygen steady-state concentrations in general.

Eqn (2) can be conceptualized as comprising five separate terms; the first term describes the amount of light entering the water body, the second term describes the fraction of that light that is attenuated in the water column by all processes, the third term is the fraction of light that is not removed from the water column by backscattering (*i.e.* the fraction of light remaining after backscattering occurs), the fourth term is the fraction of the total light absorbance that is absorbance by CDOM, and the fifth term describes the efficiency at which singlet oxygen is produced from the photons absorbed by CDOM. Eqn (2) can also be used to calculate near-surface concentrations by setting 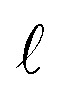 to a shallow depth (1 cm in this work). With the exception of 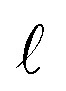 at the near-surface, the parameters that govern the terms in eqn (2) are not set values, rather they vary depending on environmental factors. Note that incident irradiance, the diffuse attenuation coefficient, and singlet oxygen quantum yields are all functions of wavelength, represented by a subscript *λ* in eqn (2). When subsequently referring to these variables in the text, the *λ* has been omitted for brevity. While *f*_backscatter_ and *f*_abs,CDOM_ are also functions of wavelength, the wavelength dependence of these parameters is not as well understood. For this reason, single values of *f*_backscatter_ and *f*_abs,CDOM_ are used here. Some of the parameters in eqn (2) also depend either implicitly or explicitly on other factors including wavelength (*λ*, nm), latitude (°N or °S), dissolved organic carbon concentration (DOC, mg_C_ L^−1^), and temporal factors such as season or time of day. Additionally, many of the parameters are interdependent. For example, light in the water column is attenuated as a function of both depth and wavelength, and the rate of attenuation depends on the DOC concentration in the water body. Finally, the only quenching process for ^1^O_2_ considered in eqn (1) is ^1^O_2_ deactivation by water (*k*_d_, s^−1^). While other ^1^O_2_ sinks exist such as reaction with pollutants, previous work has found that in most natural waters the effect of other ^1^O_2_ quenchers is negligible compared to physical deactivation by water.^11^ The value of *k*_d_ was recently updated for ^1^O_2_ deactivation in water, and the updated value of (2.76 ± 0.02) × 10^5^ s^−1^ measured at 20 °C has been used here.^28^*k*_d_ is weakly temperature dependent,^28^ but for temperatures ranging from 5 to 30 °C, subsequent calculated values of [^1^O_2_]_SS_ vary by less than 5%.

A form of eqn (2) has been used by researchers over the past 30 years.^7,29^ However, for filtered laboratory samples, decadic absorbance has typically been used in place of diffuse attenuation coefficients, and assumptions are made that negate the need for the *f*_backscatter_ and *f*_abs,CDOM_ terms (eqn S9†). When decadic absorbance is used in eqn (2) instead of the diffuse attenuation coefficient, the first two terms are often referred to as the rate of light absorbance, and are derived from the physics of light absorption.^30^ The form of eqn (2) used here accounts for light scattering and for components other than CDOM that absorb light in the water column. As such, we believe it better represents the rate of formation of ^1^O_2_ in the environment.

**Fig. 1:**
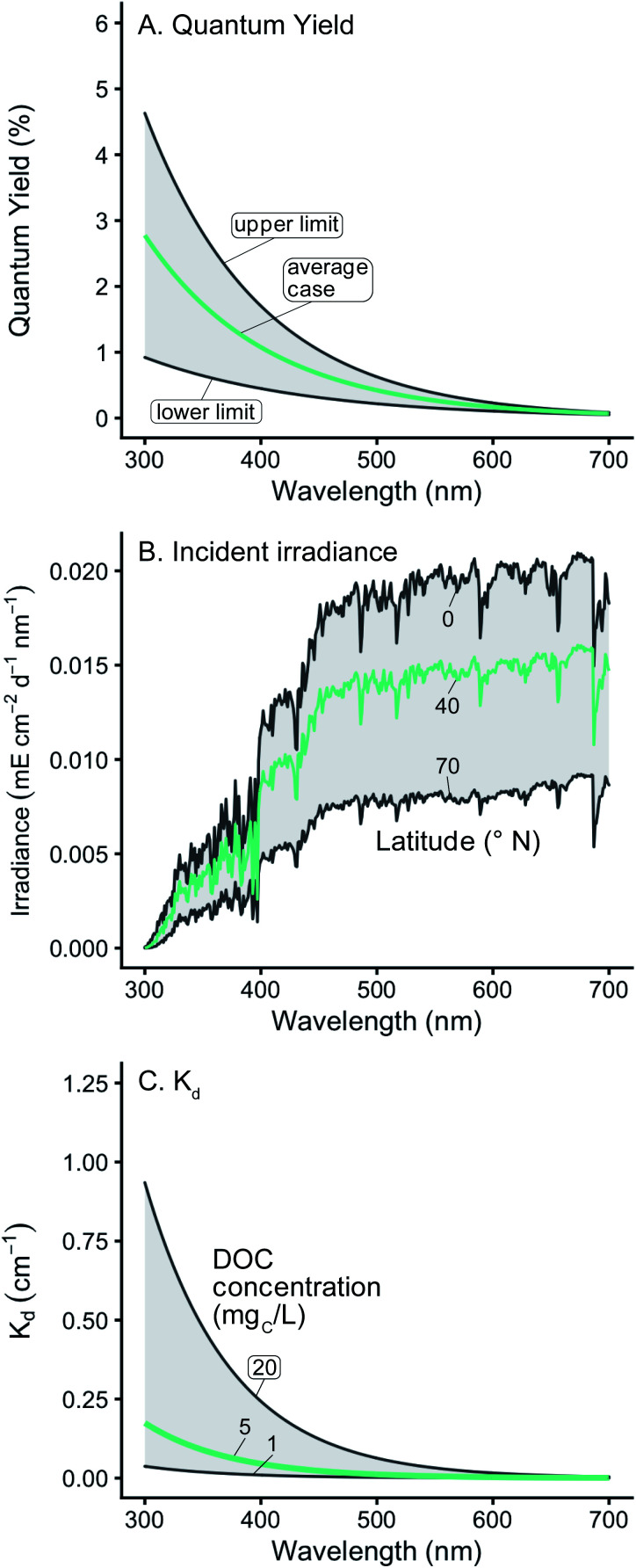
(A) Range of wavelength-dependent ^1^O_2_ quantum yields used. The shaded area represents the envelope that includes all the experimental data found in Partanen *et al.*,^31^ and the green line represents the average of the two bounds. (B) Range of incident solar irradiance spectra used. The green line shows a representative case of 40° N. (C) Range of modeled diffuse attenuation coefficients (*K*_d_) used. The green line shows a representative case of modelled *K*_d_ values using 5 mg_C_ L^−1^.

In this work the variability of [^1^O_2_]_SS_ within epilimnia and at the near-surface of representative lakes was examined. Variability in [^1^O_2_]^epi,avg^_SS_ was explored by defining a range of possible values as well as a “representative case” for epilimnion depth, latitude, DOC concentration, singlet oxygen quantum yield, backscattering fraction, and fraction of total absorbance attributable to CDOM (Table 1 and Fig. 1). Note that setting values for latitude and DOC also sets the *I*_0_ values and *K*_d_ values, respectively. The confidence column in Table 1 gives a qualitative assessment of the data available (both quantity and quality) for each of the parameters, and will be discussed in the following sections. To investigate which parameter had the largest effect on [^1^O_2_]^epi,avg^_SS_, each parameter was varied individually over the range of possible values, while the remaining parameters were held constant at their representative value.

**Table 1:** Range of values for key parameters. Values used as a representative case are also reported

Parameter	Representative value	Minimum value	Maximum value	Confidence
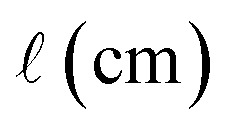	900	200	2000	Medium–high
Latitude (°N)	40	70	0	High
DOC (mg_C_ L^−1^)	5	1	20	Medium–high
*Φ*_Δ,*λ*_ value at 365 nm (%)	1.5	0.58	2.4	Medium–low
*f*_backscatter_	0.03	0.005	0.07	Medium–low
*f*_abs,CDOM_	0.7	0.3	0.9	Low

**Table 2:** [^1^O_2_]^epi,avg^_SS_ values for a range of epilimnion depths and latitudes. Estimated using DOC = 5 mg_C_ L^−1^, the representative case for *Φ*_Δ_ relationship, and average annual incident irradiance

	[^1^O_2_]_SS_,^epi,avg^ (10^−16^ M)							
	Latitude (° N)							
Epilimnion depth (m)	0	10	20	30	40	50	60	70
2	32	31	29	27	23	20	16	13
3	22	22	20	19	16	14	11	8.8
4	17	17	16	14	13	10	8.4	6.8
5	14	14	13	12	10	8.5	6.8	5.5
6	12	11	11	9.8	8.6	7.2	5.8	4.6
7	10	9.8	9.3	8.5	7.4	6.2	5	4
8	8.8	8.6	8.2	7.5	6.5	5.5	4.4	3.5
9	7.9	7.7	7.3	6.7	5.8	4.9	3.9	3.1
10	7.1	7	6.6	6	5.3	4.4	3.5	2.8
11	6.5	6.3	6	5.5	4.8	4	3.2	2.6
12	5.9	5.8	5.5	5	4.4	3.7	3	2.4
13	5.5	5.4	5.1	4.7	4.1	3.4	2.7	2.2
14	5.1	5	4.7	4.3	3.8	3.2	2.5	2
15	4.8	4.7	4.4	4.1	3.5	3	2.4	1.9
16	4.5	4.4	4.2	3.8	3.3	2.8	2.2	1.8
17	4.2	4.1	3.9	3.6	3.1	2.6	2.1	1.7
18	4	3.9	3.7	3.4	3	2.5	2	1.6
19	3.8	3.7	3.5	3.2	2.8	2.3	1.9	1.5
20	3.6	3.5	3.3	3.1	2.7	2.2	1.8	1.4

### Data sources

#### Epilimnion depth

Understanding the physical characteristics of global surface waters is a prerequisite to determining the behavior of ^1^O_2_ within those waters. Recently, Qin *et al.* compiled a Global Lakes Database of 573 lakes from around the world that includes mean and maximum lake depth and modelled epilimnion depth.^32^ In the Global Lakes Database, epilimnion depth is modelled based on lake area using a relationship from Hanna:^33^3
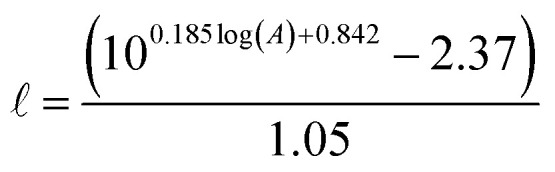
where 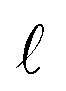 is the epilimnion depth in *m*, and *A* is the lake area in km^2^.

Using this empirical relationship, it is possible to calculate a theoretical epilimnion depth that is greater than the maximum depth of the lake. This occurred for 13% of the lakes in the Global Lakes Database. We assumed a lake to be fully mixed if its theoretical epilimnion depth was greater than the maximum lake depth. In these cases the maximum lake depth was used in place of the epilimnion depth. Summary plots and statistics for the 573 lakes are available in the ESI (Fig. S1 and Table S1†). Briefly, the average epilimnion depth is 9 m, and 96% of lakes have an epilimnion depth that falls between 2 and 20 m.

#### Quantum yield relationship

^1^O_2_ quantum yields are wavelength-dependent, but it is common to simplify this relationship to a single value. To more accurately reflect environmental conditions, a range of possible wavelength-dependent *Φ*_Δ_ relationships was modelled based on measured *Φ*_Δ_ relationships for Suwannee River NOM (SRNOM), Pony Lake Fulvic Acid (PLFA), Suwannee River water, and Étang de la Gruère water reported in Partanen *et al.* (Fig. 1A).^31^ Additional details about how this range was modelled and how the experimental and modelled data compare can be found in the ESI and in Fig. S3.† To compare the impact of using single *Φ*_Δ_ values (not wavelength-dependent) *versus* wavelength-dependent *Φ*_Δ_ values on [^1^O_2_]^epi,avg^_SS_, *Φ*_Δ_ values for SRNOM and PLFA were examined in detail. For this analysis, wavelength-dependent relationships for SRNOM and PLFA were modelled using bi-exponential fits to experimental data^31^ (Fig. S4†).

**Fig. 2:**
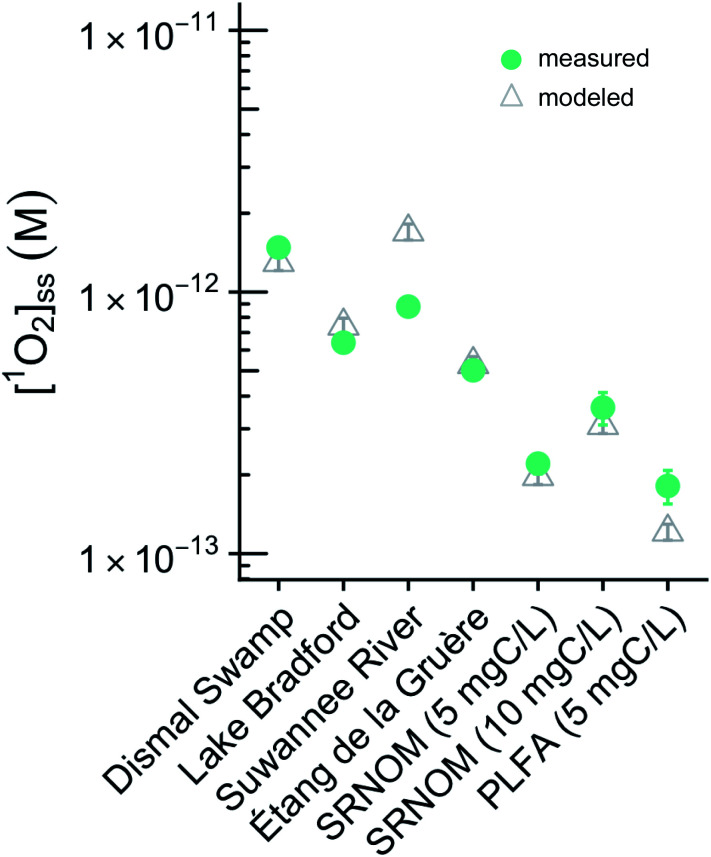
Comparison between measured and calculated values for natural water and organic matter isolate samples. Error bars on measured values represent one standard deviation of triplicate measurements. Error bars on calculated values represent the variability in experimentally quantified UVA lamp spectral irradiance.

**Fig. 3:**
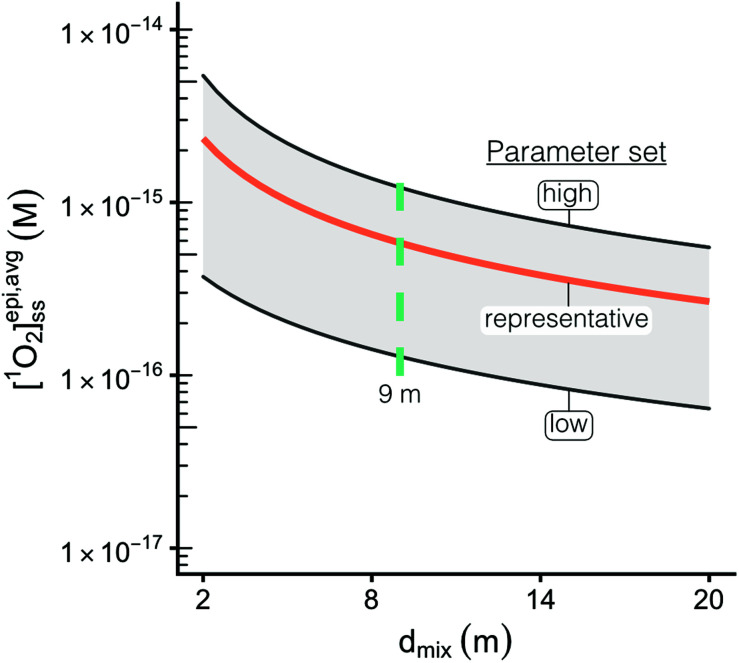
Range of [^1^O_2_]^epi,avg^_SS_ values as a function of epilimnion depth for the “representative set” of parameters (orange line). The shaded region represents the parameters that produce the highest [^1^O_2_]^epi,avg^_SS_ values (DOC = 20 mg_C_ L^−1^, latitude = 0°, upper limit of modelled *Φ*_Δ_ relationships) and the lowest [^1^O_2_]^epi,avg^_SS_ values (DOC = 1 mg_C_ L^−1^, latitude = 70°N, lower limit of modelled *Φ*_Δ_ relationships). Green dotted line shows the mean epilimnion depth.

**Fig. 4:**
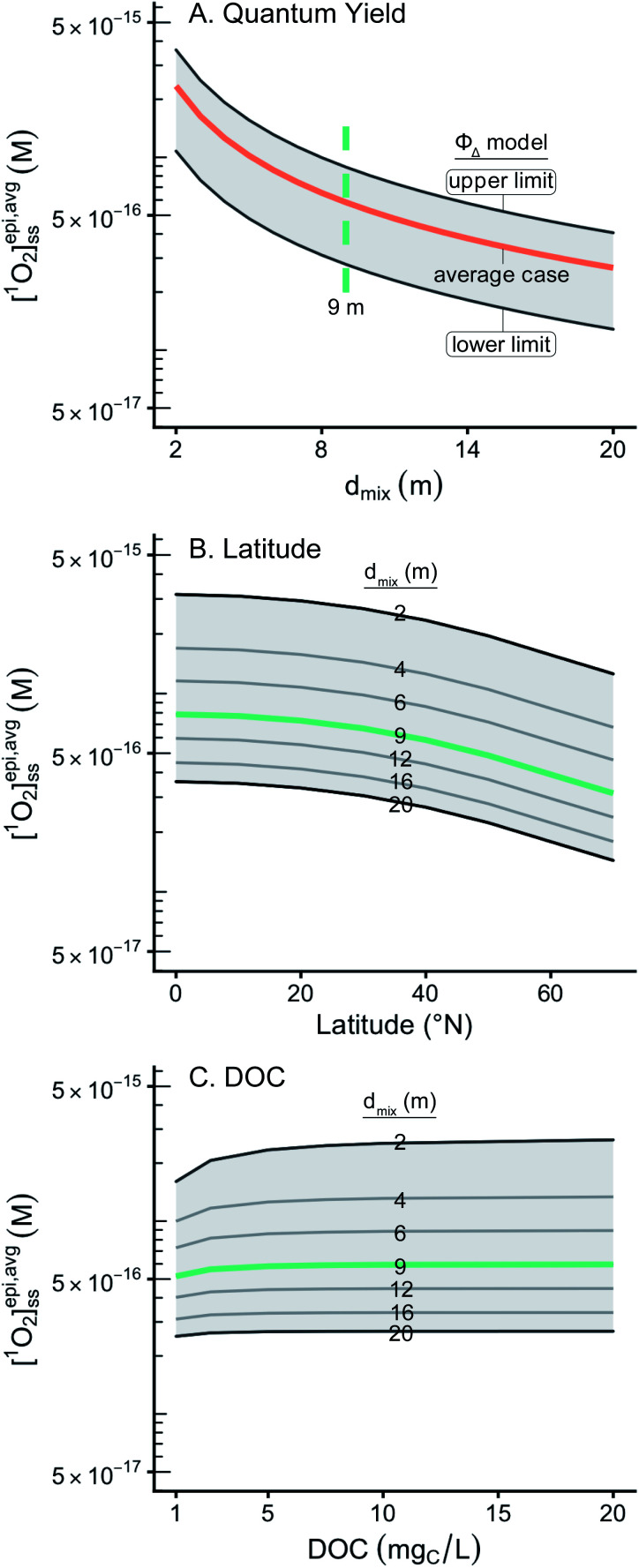
Impact of variable input parameters on [^1^O_2_]^epi,avg^_SS_ as a function of epilimnion depth. In panels (A, B and C), one parameter is varied, while the rest of the parameters are held constant at their “representative case” values. (A) Range of possible [^1^O_2_]^epi,avg^_SS_ values for different modelled *Φ*_Δ_ relationships. Shaded region represents the upper and lower limits of the *Φ*_Δ_ relationships, orange line represents the average of this range. The green line represents the mean epilimnion depth in the Global Lakes Database (9 m). (B) Range of possible [^1^O_2_]^epi,avg^_SS_ values for latitudes ranging from the equator to 70°N for multiple epilimnion depths. Shaded region represents 2–20 m epilimnion depths, green line represents the mean epilimnion depth (9 m). (C) Range of possible [^1^O_2_]^epi,avg^_SS_ values for DOC concentrations ranging from 1 to 20 mg_C_ L^−1^ for multiple epilimnion depths. Shaded region represents 2–20 m epilimnion depths, green line represents the mean epilimnion depth (9 m).

**Fig. 5:**
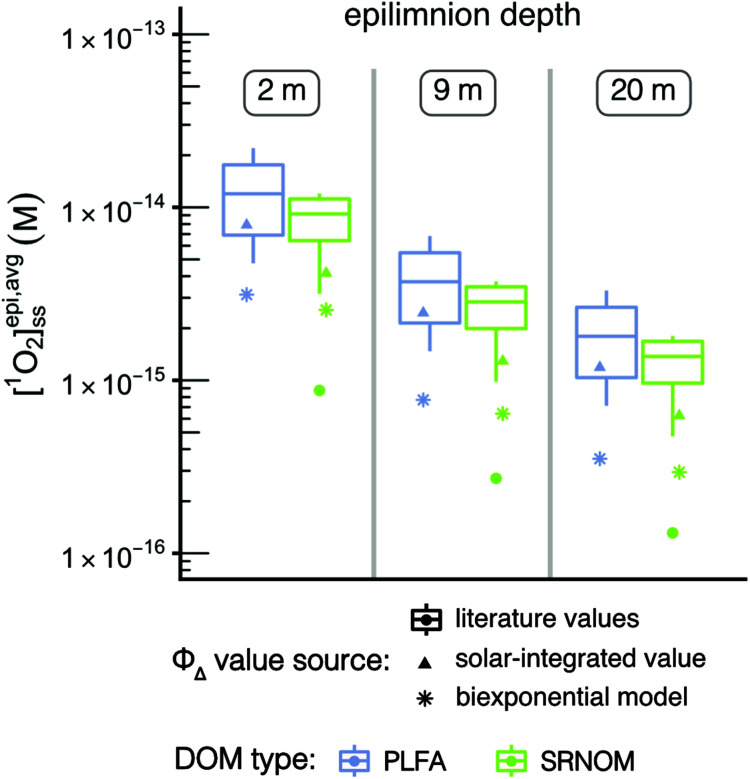
[^1^O_2_]_SS_ values calculated using *Φ*_Δ_ values from PLFA (blue sides) or SRNOM (green sides) using either a range of literature values (box plots, where the lower and upper edges represent the first and third quartiles, the middle line represents the median, the ends of the whiskers represent the largest or smallest value no further than 1.5× the interquartile range, and the circle symbols represent outliers in the dataset), the best available solar-integrated *Φ*_Δ_ value for either PLFA or SRNOM (triangles), or a wavelength-dependent *Φ*_Δ_ relationship fit to a biexponential model for PLFA or SRNOM (stars).

Note that the delta symbol in the notation for ^1^O_2_ quantum yield (*Φ*_Δ_) comes from the term symbol for the lowest energy singlet excited state of molecular oxygen (*i.e.*^1^Δ_g_). The delta symbol has become a shorthand in the literature for ^1^O_2_ (^1^Δ_g_).

#### Incident irradiance

Solar irradiance varies throughout the day and year and as a function of latitude. Published reference solar spectra for the average daily irradiance during solstices and equinoxes, which were modelled using the Simple Model of the Atmospheric Radiative Transfer of Sunshine (SMARTS) and validated against high-resolution spectroradiometers, were averaged to obtain annual reference spectra.^34^ The temporal variability of irradiance was averaged because the goal was to find representative [^1^O_2_]_SS_ regardless of the time of year and for degradation processes that generally proceed at timescales of weeks or longer. The variability in irradiance due to latitude was retained within the model to quantify the impact of geographic location (Fig. 1B). The reference solar spectra do not account for reflectance from the water surface; therefore, the impact of reflectance was also investigated but was found to be small (*i.e.*, ≤15% difference in irradiance at all wavelengths, see the ESI† for details and calculations).

#### Diffuse attenuation coefficients

There have been many attempts to model both *K*_d_ and absorbance spectra based on the chemical or optical properties of a water body. In this work, we use a relationship where *K*_d_ spectra are modelled using DOC (mg_C_ L^−1^) and wavelength as the only inputs (eqn (4)). This relationship was developed from measured values of *K*_d_ at narrow-band wavelengths within the UV range using data from 59 lakes where the DOC ranged from 0.24 to 23.5 mg_C_ L^−1^.^35^ Since the *K*_d_ model was developed using UV wavelengths, its results are most reliable within this wavelength range, and its extension into the visible range should be viewed with more caution.

4 *K*_d_ = exp(−0.01347*λ* + 5.36(DOC)^0.157^)

#### Backscattering fraction and fraction of CDOM absorbance

The range of values used for *f*_backscatter_ and *f*_a,CDOM_ come from the literature, where much of the available data is from marine environments, and has been measured at visible wavelengths. The impact of *f*_backscatter_ and *f*_a,CDOM_ on values of [^1^O_2_]^epi,avg^_SS_ was investigated (Fig. S11†), and since there is a lack of data for a wide variety of lakes at UV wavelengths, representative or average values were chosen for subsequent analysis.

For *f*_backscatter_, a range of single values (not wavelength-dependent) were investigated,^36–40^ and a representative value of 0.03 was chosen for analysis. Whether *f*_backscatter_ is wavelength-dependent is somewhat controversial, though recent research suggests that the fraction decreases with increasing wavelength.^37,41^ However, very little wavelength-dependent *f*_backscatter_ data exists in the literature, especially in the UV region.

*f*_a,CDOM_ is most often measured over photosynthetically active radiation wavelengths (400–700 nm)^42–46^ or at 440 nm,^47–50^ with only a few measurements made within the UV region.^51–53^ As with *f*_backscatter_, wavelength-dependent information for *f*_a,CDOM_ is sparse. The value used for *f*_a,CDOM_ has a large impact on [^1^O_2_]^epi,avg^_SS_ (Fig. S11†), but since this parameter is not well characterized for a variety of lake types, an average value for measurements in the UV range (0.7) was used throughout this work.

### Environmental half-life calculations

The estimated ranges of values for [^1^O_2_]^epi,avg^_SS_ were used to calculate half-lives (*t*_1/2_) of chemical compounds in the epilimnia of lakes due to reaction with ^1^O_2_ using eqn (5):5
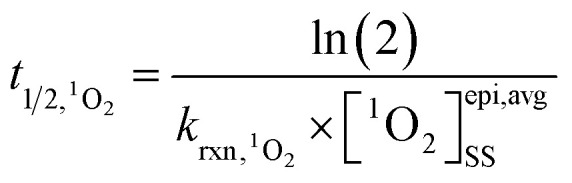
where 
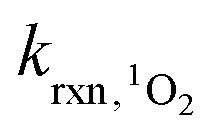
 is the bimolecular rate constant of a compound reacting with ^1^O_2_ (M^−1^ s^−1^).

### Relating test tube [^1^O_2_]_SS_ to surface [^1^O_2_]_SS_

Values of [^1^O_2_]_SS_ determined for natural water samples in test tubes in the laboratory (referred to as the “test tube case”) can be similar to environmental near-surface [^1^O_2_]_SS_ values because the depth in both cases is very shallow (*i.e.*, 1 cm), though in order for this equivalency to hold, the incident irradiance in both the laboratory and the near-surface environment must be the same. Test tube measurements are important because near-surface values of [^1^O_2_]_SS_ represent the conditions described in testing guidelines such as the one published by the US EPA^54^ and are also representative of most of the literature values of [^1^O_2_]_SS_.^13,18,21–23^

Test tube [^1^O_2_]_SS_ values for seven surface waters and organic matter isolates were experimentally determined in a Rayonet photoreactor using furfuryl alcohol as a probe molecule for ^1^O_2_. Experimental details can be found in the ESI.† Values of [^1^O_2_]_SS_ for these seven samples were then independently estimated using eqn (1) and S9,† where the irradiance parameter is the spectral output of the lamps used in the laboratory (Fig. S15†), the absorbance spectra of the water samples were measured by a UV-vis spectrophotometer (Fig. S13†), and 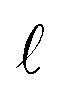 is equal to the average pathlength of the test tube (*i.e.*, 1 cm). *Φ*_Δ_ values for the samples are from Partanen *et al.*^31^ (Table S5†), and were independently measured using the ^1^O_2_ phosphorescence method, rather than through steady-state photolysis. The experimentally determined [^1^O_2_]_SS_ values were then compared with the independently calculated values. In this analysis eqn S9† was used instead of eqn (2) because experimentally determined absorbance spectra for the samples were available, and because the samples were filtered, meaning that the impact of particulates was assumed to be negligible. Details on the collection and characterization of these samples can be found in the ESI.†

For the test tube case, differences between values of [^1^O_2_]_SS_ calculated using eqn 1 and S9,† and values of [^1^O_2_]_SS_ measured in the laboratory range from 5 to 64% (Fig. 2). The experimental values are between 1.8 × 10^−13^ (±2.7 × 10^−14^) and 1.5 × 10^−12^ (±1.0 × 10^−13^) M, whereas values calculated using eqn 1 and S9† range from 1.2 × 10^−13^ (±8.5 × 10^−15^) to 1.7 × 10^−12^ (±1.2 × 10^−13^) M.

Reference *Φ*_Δ_ values for the seven samples were measured independently in previous work using the ^1^O_2_ phosphorescence method,^31^ and were subsequently used in calculations of [^1^O_2_]_SS_. These values were used so that the calculated values of [^1^O_2_]_SS_ would not rely on *Φ*_Δ_ values determined from the steady-state photolysis experiments performed for the test-tube case. However, it is also possible to calculate *Φ*_Δ_ values from the steady-state photolyses used to experimentally determine [^1^O_2_]_SS_ values (see the ESI† for details). The difference between *Φ*_Δ_ values determined using steady-state photolysis and the reference *Φ*_Δ_ values from Partanen *et al.* for each of the samples closely tracks the difference between experimentally measured and calculated [^1^O_2_]_SS_ values, indicating that this parameter is the source of much of the variability. There are many potential sources of error when measuring *Φ*_Δ_ values,^55^ and it is likely experimental error that leads to the difference between experimentally measured *Φ*_Δ_ values and reference *Φ*_Δ_ values in this analysis, rather than an incorrect [^1^O_2_]_SS_ estimation framework. Overall, our approach estimates [^1^O_2_]_SS_ values that are in good agreement with experimentally measured [^1^O_2_]_SS_ values for the test-tube case.

## Results and discussion

For a given epilimnion depth, the [^1^O_2_]^epi,avg^_SS_ can vary by over an order of magnitude depending on the set of input parameters (*Φ*_Δ_ relationship, latitude, and DOC concentration) (Fig. 3). Similarly, varying epilimnion depth from 2–20 m (accounting for 96% of lakes within the Global Lakes Database^32^) causes a decrease in [^1^O_2_]^epi,avg^_SS_ by a factor of 8.8 (Table 3). The *Φ*_Δ_ relationship used and the latitude of the lake have the next largest impacts (Table 3, Fig. 4A and B). Finally, while DOC concentrations have a substantial effect on near-surface [^1^O_2_]_SS_, at epilimnion depths below 2 m increasing the DOC concentration from 1–20 mg_C_ L^−1^ has only a small effect on [^1^O_2_]^epi,avg^_SS_ (Table 3 and Fig. 4C). Data tables of [^1^O_2_]^epi,avg^_SS_ for a range of epilimnion depths and latitudes and for three sets of input parameters can be found in Tables 2 and S6.† The results shown in Table 3, Fig. 3 and 4 will be discussed in the following sections.

**Table 3:** Impact of different parameters on the variability of [^1^O_2_]^epi,avg^_SS_. One parameter is varied at a time, while the rest are held in the representative case (5.0 mg_C_ L^−1^, 40° N, average *Φ*_Δ_ as a function of wavelength relationship, 9 m mixed layer depth)

Parameter being varied	Range of values (test set)	[^1^O_2_]^epi,avg^_SS_ (×10^−16^ M)	Factor difference between max and min values
Epilimnion depth (m)	2.0	23	8.8
	20	2.7	
QY (%)	Lower limit^a^	2.8	3.2
	Upper limit^a^	9.0	
Latitude (° N)	0	7.9	2.5
	70	3.1	
DOC (mg_C_ L^−1^)	1.0	5.2	1.2
	20.0	6.0	

a see Fig. 1A.

The range of [^1^O_2_]_SS_ values often quoted in the literature is 10^−14^ to 10^−12^ M.^7,13,56^ This range explicitly refers to the top layer (<1 cm) of a sunlit lake under ideal conditions (noon, summer day), and not to the epilimnion or to less ideal conditions. We propose using a range of 6 × 10^−17^ to 5 × 10^−15^ M to represent values of [^1^O_2_]_SS_ within the mixed layer of lakes. This range represents the top left-most point (epilimnion depth = 2 m, DOC = 20 mg_C_ L^−1^, Latitude = 0°, upper limit of modelled *Φ*_Δ_ relationships) to bottom right-most point (epilimnion depth = 20 m, DOC = 1 mg_C_ L^−1^, Latitude = 70°N, lower limit of modelled *Φ*_Δ_relationships) of Fig. 3.

### Epilimnion depth and lake depth

The epilimnion depth has a large impact on depth-averaged values of [^1^O_2_]_SS_ within a lake, causing variation of close to a factor of 9 over epilimnion depths between 2 and 20 m. The reason for the large variability in [^1^O_2_]^epi,avg^_SS_ is because once all of the photons entering the water column are absorbed (Fig. S7†), no additional ^1^O_2_ can be produced. Therefore, as the epilimnion depth increases, the same amount of ^1^O_2_ is diluted over a larger volume of water. While many [^1^O_2_]_SS_ values reported in the literature are valid for the near-surface of lakes, the average epilimnion depth within the Global Lakes Database is 9 m. Because the two scenarios substantially differ in terms of [^1^O_2_]_SS_, it is important to ensure that values for the near-surface are not conflated with depth-averaged values.

It is also important to note that the equation representing the rate of formation of ^1^O_2_ differs depending on whether the rate is being calculated for a specific lake depth or is averaged over the epilimnion depth. Eqn (1) and (2) presented above are used to calculate an average concentration of ^1^O_2_ within the epilimnion of a lake, whereas eqn (6) below is used to calculate the rate of formation of ^1^O_2_ at a given depth (*i.e.*, assuming no mixing).6
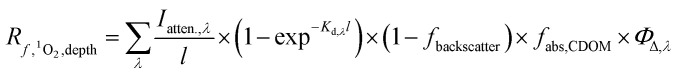

7 *I*_atten.,*λ*_ = *I*_0,*λ*_ × exp^−*K*_d,*λ*_*d*^

where 
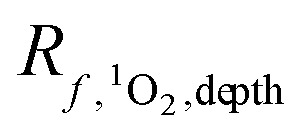
 is the rate of formation of ^1^O_2_ in a water body at a given depth (M s^−1^), *I*_atten.,*λ*_ is the attenuated irradiance (mmol photons cm^−2^ s^−1^ nm^−1^), *l* is the pathlength (1 cm intervals were used in this work), *d* is the depth of interest (cm) at which the attenuated irradiance is being calculated, and *K*_d,*λ*_, *f*_backscatter_, *f*_abs,CDOM_, *Φ*_Δ,*λ*_, and *I*_0,*λ*_ are defined above.

There are two main differences between eqn (2) and (6). First, when calculating 
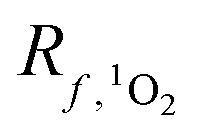
 at a particular depth, the attenuation of light due to the overlying water column must be taken into account using eqn (7), where *d* is the depth at which the attenuated irradiance is being calculated. Second, in eqn (1), 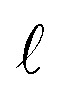 is the epilimnion depth, whereas in eqn (6)*R*_f_ is being calculated within subsequent thin layers of water, so *l* is a constant value. Note that where water bodies are not considered well-mixed, such as below the epilimnion where vertical mixing is slow, the [^1^O_2_]_SS_ varies as a function of depth (*i.e.*, according to equation (6)). A graphical representation of the difference between [^1^O_2_]_SS_ as a function of the depth of a water body and [^1^O_2_]^epi,avg^_SS_ as a function of well-mixed epilimnion depth can be seen in Fig. S2.†

The calculated [^1^O_2_]^epi,avg^_SS_ values are only applicable to the epilimnia of lakes and do not apply to the hypolimnia of the lakes. Therefore, calculated lifetimes of ^1^O_2_-reactive compounds are also only valid for the epilimnion. If such a compound is not present within the epilimnion (*e.g.* introduced into the lake *via* groundwater infiltration), then it is not possible to estimate the pollutant lifetime based on the [^1^O_2_]^epi,avg^_SS_ values presented here.

### Quantum yield relationships

The particular *Φ*_Δ_ relationship used in calculations of 
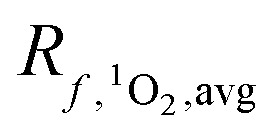
 impacts values of [^1^O_2_]^epi,avg^_SS_ by a factor of 3.2 (Fig. 4A) between the upper and lower limits of the modelled *Φ*_Δ_ relationship (see Table 1 and Fig. 1A), but unlike epilimnion depth and latitude, this variability is in part due to limited experimental data rather than innate environmental variability. The range of *Φ*_Δ_ values used here was modelled based on experimentally determined wavelength-dependent relationships of *Φ*_Δ_ that we believe are currently the best available data^31^ (Fig. S3†). However, much remains unknown about the wavelength dependence of *Φ*_Δ_, and more information could reduce the uncertainty in this parameter. Specifically, having the ability to correlate a wavelength-dependent *Φ*_Δ_ relationship to different types of water bodies would help to reduce the variability attributable to this parameter. In addition, the available wavelength-dependent experimental data only includes organic matter from lakes and rivers, and does not include other types of organic matter such as effluent organic matter or marine organic matter. Since these types of organic matter are known to have different single-value *Φ*_Δ_ compared to lake or river organic matter,^21,57^ they may also have different wavelength-dependent *Φ*_Δ_ relationships.

Values of *Φ*_Δ_ are also known to increase with increasing oxygen concentration within the range of 0.96–9.3 mg_O_2__ L^−1^.^58^ Since the oxygen concentration within surface waters varies both seasonally and diurnally as a nonlinear function of temperature and phytoplankton activity,^59^ one would expect *Φ*_Δ_ values within lakes to be higher when the oxygen saturation is higher. *Φ*_Δ_ values will mainly be affected when water temperatures are above 19 °C, as below 19 °C *Φ*_Δ_ has been found to be constant with respect to [O_2_].^59^ While it is clear that *Φ*_Δ_ can vary as a function of [O_2_], we believe that the range of values for *Φ*_Δ_ used here capture this variability.

Since it is common to simplify the *Φ*_Δ,*λ*_ relationship to a single value across the wavelength range being studied, the impact of using single values for *Φ*_Δ_ on the calculated values of [^1^O_2_]^epi,avg^_SS_ was explored. Two different sources of single values for *Φ*_Δ_ were investigated: (1) solar-integrated *Φ*_Δ_ values and (2) the range of *Φ*_Δ_ values available from the literature. Solar-integrated *Φ*_Δ_ values for SRNOM (1.0%) and PLFA (1.9%) were taken from Partanen *et al.*^31^ and were calculated by integrating the wavelength-dependent *Φ*_Δ_ relationships for these isolates over the entire solar spectrum. The range of *Φ*_Δ_ values obtained from the literature were 0.21–2.89% for SRNOM (*n* = 15) and 1.14–5.29% for PLFA (*n* = 10).^17^ These literature values were measured using a solar simulator or xenon lamp to represent solar irradiance. [^1^O_2_]^epi,avg^_SS_ values were calculated from the single values for *Φ*_Δ_, and were compared to [^1^O_2_]^epi,avg^_SS_ values calculated using biexponential models of wavelength-dependent *Φ*_Δ_ data for SRNOM and PLFA (Fig. S4†).

Values of [^1^O_2_]^epi,avg^_SS_ calculated with wavelength-dependent *Φ*_Δ_ relationships are consistently lower than any single *Φ*_Δ_ value (Fig. 5, stars compared to box plots and triangles). Of the non-wavelength-dependent *Φ*_Δ_ values used for PLFA and SRNOM, the [^1^O_2_]^epi,avg^_SS_ values calculated using solar-integrated *Φ*_Δ_ values (triangles) are below the median of those calculated using the range of values reported in the literature (boxplots). Note that outliers in the boxplots are represented by dots. This analysis shows that if a non-wavelength-dependent value for *Φ*_Δ_ is used in calculations of [^1^O_2_]^epi,avg^_SS_, it generally results in higher estimations of [^1^O_2_]^epi,avg^_SS_ at any epilimnion depth, although there is one outlier literature *Φ*_Δ_ value that would predict lower [^1^O_2_]^epi,avg^_SS_. This reinforces the need for more complete experimental data on *Φ*_Δ_ wavelength-dependent relationships.

### Latitude and other factors affecting incident irradiance

Variability in incident irradiance comes from multiple sources, some of which are more straightforward to account for than others. Latitude is the largest source of variability in the incident irradiance but is accounted for in the reference irradiance spectra. The reference spectra themselves also have some uncertainty, as they tend to overestimate the values measured by spectroradiometers.^34^ The calculated [^1^O_2_]^epi,avg^_SS_ in a lake with an average epilimnion depth (9 m) was found to vary by a factor of 2.5 depending on whether calculations of 
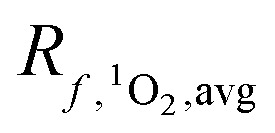
 use incident irradiance spectra from the equator or from above the Arctic circle (Fig. 4B). In contrast, reflectance of light from the surface of a water body decreases the number of photons entering the water column by 4.5 to 15% for latitudes between 0 and 70° N (Table S2 and Fig. S5; † details on the reflectance calculations can also be found in the ESI†).

The season also impacts the incident irradiance, and in this work average annual irradiance spectra were used, as this likely gives a more accurate representation of the amount of ^1^O_2_ in the water column on timescales relevant to pollutant lifetimes. These annual average spectra were calculated by averaging day-averaged spectra from the solstices and equinoxes. When estimating environmental concentrations of PPRIs such as ^1^O_2_, it is common to use a summer irradiance spectrum (either day-averaged or solar noon) as the input for incident irradiance,^18–20^ but these irradiance spectra represent a “best-case scenario” in terms of the number of photons entering the water body. Using sunny summer day or June 21^st^ solar noon spectra results in [^1^O_2_]^epi,avg^_SS_ values that are higher by a factor of 1.5 and 4.4, respectively, when compared to using average annual irradiance spectra (Fig. S6†).

Another factor that can impact incident irradiance is the effect of clouds, which depends on the extent of cloud cover, the type of cloud, and seasonal and location variability. Some progress has been made in developing empirical models that relate the ratio of measured irradiance under cloudy conditions and the estimated irradiance under cloudless conditions^60^ to the fraction of cloud coverage.^61–63^ However, not all of these models capture the impact of different types of clouds and many require location-specific information. Cloud cover will impact the incident irradiance entering a lake or river, but because the impact is so location specific, it is not accounted for in the calculations of [^1^O_2_]^epi,avg^_SS_ presented here.

Overall, reflectance off the water surface, cloud cover, and using average annual irradiance instead of summer day or solar noon values decrease the incident irradiance, leading to lower [^1^O_2_]^epi,avg^_SS_ values in the epilimnia of lakes. The proper magnitude of this decrease must await future studies that give better estimates of these effects on the incident irradiance entering a surface water.

### DOC concentration has a limited impact on depth-averaged [^1^O_2_]^epi,avg^_SS_

Changes in the DOC concentration in a lake have an impact on the modelled diffuse attenuation coefficients used in this work (Fig. 1C), but [^1^O_2_]^epi,avg^_SS_ changes by only 14% over a range of 1 to 20 mg_C_ L^−1^ at an epilimnion depth of 9 m (Fig. 4C). Once all the photons entering a water body are absorbed, the change in [^1^O_2_]^epi,avg^_SS_ over a range of DOC concentrations decreases as epilimnion depth increases due to the increase in dilution volume. Thus, for a 20 m epilimnion, the change in [^1^O_2_]^epi,avg^_SS_ is only 6% over the same DOC range. The extreme case occurs at the near-surface of lakes, where DOC concentration has a large impact on [^1^O_2_]_SS_. This special case is discussed in the section “Estimations of [^1^O_2_]_SS_ at the near-surface of lakes” below.

DOC concentration has such a limited effect on [^1^O_2_]^epi,avg^_SS_ within the epilimnion of lakes because once all the photons entering the water column are absorbed by DOM, increasing the DOC has no further effect on ^1^O_2_ production. The depth at which the majority of photons are absorbed in the water column depends on the DOC concentration and on the wavelength of light (see Fig. S7†), but light at all but the longest wavelengths (>500 nm) is completely attenuated by 4 m, even for lakes containing only 1 mg_C_ L^−1^. Therefore, for most lakes, the same number of photons are absorbed in the epilimnion irrespective of the DOC concentration within the lake, and thus for the epilimnion of stratified lakes or for shallow well mixed lakes, the [^1^O_2_]^epi,avg^_SS_ is not greatly impacted by DOC concentration.

### Diffuse attenuation coefficients and absorbance spectra

In this work, modelled diffuse attenuation coefficients (*K*_d_) were used to represent the absorbance and scattering of light in the water column (Fig. 1C).^35^*K*_d_ is theoretically better for modelling [^1^O_2_]^epi,avg^_SS_ in real surface waters compared to decadic absorbance because it takes into account all light attenuation processes within the water column. However, to accurately calculate [^1^O_2_]^epi,avg^_SS_ using *K*_d_ values, both the backscattering fraction and the fraction of total light absorbance that is specific to CDOM must also be known.

The *K*_d_ relationship used in this work (eqn (4)) is a function of DOC concentration, and was developed using data from lakes in a variety of different locations and spanning a wide range of DOC concentrations.^35^ As such, we believe it provides a reasonable estimate of *K*_d_ values within lakes, though researchers have found that *K*_d_ values measured in surface waters using radiometers sometimes deviate from *K*_d_ values modelled based on DOC concentrations.^44,64–66^ These differences have been attributed to attenuation caused by scattering or absorbance by particulate components, which are not explicitly accounted for in attenuation models that use DOC as the only input parameter. Scattering and particle absorbance can account for anywhere from 20 to ∼70% of light attenuation depending on factors such as turbidity and presence of algal blooms.^35,44,64,65^ Most data used to develop *K*_d_ relationships is specific to a certain lake type or location, meaning that relationships developed for high-turbidity lakes will be different than those developed for clear, low DOC waters. Thus, if site-specific *K*_d_ values are known, estimates of [^1^O_2_]^epi,avg^_SS_ could be improved for a given waterbody.

Measured decadic absorbance spectra of filtered water samples are most often used in laboratory measurements of [^1^O_2_]_SS_, so the impact of using measured absorbance instead of modelled *K*_d_ values in calculations of [^1^O_2_]^epi,avg^_SS_ was investigated (Fig. S9†). The [^1^O_2_]^epi,avg^_SS_ calculated using absorbance spectra measured in the lab was found to be a factor of 1.5 higher than that calculated using modelled *K*_d_ values, for an epilimnion depth of 9 m and a DOC concentration of 5 mg_C_ L^−1^. Note that the equations for calculating the rate of formation of ^1^O_2_ are different if one is using a base e attenuation/absorption coefficient (*e.g.*, *K*_d_) or a base 10 coefficient (*e.g.*, *α*) (see eqn S9†). The difference in the equation for rate of formation of ^1^O_2_ is due to the fact that filtered water samples are most often used in laboratory measurements of [^1^O_2_]_SS_, leading to assumptions that all of the absorbance in the sample is due to CDOM and that there is no particle scattering. While eqn S9† may be valid for laboratory measurements, it does not capture the complexity of light attenuation processes within the water column. As such, we believe that using *K*_d_ values instead of decadic absorbance provides more accurate estimates for [^1^O_2_]^epi,avg^_SS_.

In some photochemistry models, absorbance spectra are estimated using DOC values when measured absorbance spectra are not available.^24^ We investigated the use of modelled absorbance spectra in place of measured absorbance spectra and found that in general, the modelled absorbance relationships used in the APEX model do not well represent the absorbance spectra from 7 samples of surface waters from Switzerland and the U.S. and one organic matter isolate (Fig. S8 and S10†). While this does not have a large impact on depth-averaged [^1^O_2_]^epi,avg^_SS_, as discussed above, it would have a significant effect on near-surface estimates. Experimentally obtained absorbance spectra can also be used as an input parameter, but while these spectra are more accurate for a single water body, the results may not be generalizable to other lakes. We believe that using modelled *K*_d_ values in calculations of [^1^O_2_]_SS_ gives results that are more representative of environmental systems by accounting for light scattering and absorbance by other water constituents as well as absorbance by CDOM and that they are the most generalizable to lakes globally.

### *f*_backscatter_ and *f*_abs,CDOM_

The *K*_d_ term used in eqn (2) accounts for all light attenuation, including absorbance by water, CDOM, phytoplankton, and non-algal particles, as well as light scattering.^42^ Since we assume that only light absorbance by CDOM leads to the formation of ^1^O_2_, the fraction of light backscattered out of the water column (*f*_backscatter_) and the fraction of total absorbance attributable to CDOM (*f*_abs,CDOM_) must be taken into account.

Values for *f*_backscatter_ are typically measured in marine environments, though some values exist for lakes. Literature values range from 0.005–0.07, depending on the type of water body.^36–40^ While there is some debate about the importance or even the existance of the wavelength-dependence of *f*_backscatter_, variability due to different types of water bodies is likely larger than variability due to wavelength.^36,37^ Varying *f*_backscatter_ between 0.005 and 0.07 has a limited impact on [^1^O_2_]^epi,avg^_SS_ (Fig. S11†). More data on *f*_backscatter_ in lakes, especially over UV wavelengths, could result in a more accurate value or the development of a wavelength-dependent relationship for *f*_backscatter_. However, the variability in other parameters such as *Φ*_Δ_ and *f*_abs,CDOM_ has a much greater impact on [^1^O_2_]^epi,avg^_SS_, and should therefore be the focus of future data collection efforts.

The range of values for *f*_abs,CDOM_ (0.3–0.9) is much greater than for *f*_backscatter_,^42–53^ leading to a large impact on [^1^O_2_]^epi,avg^_SS_ (Fig. S11†). However, the values in this range are not equally realistic for the environments considered in this work. Firstly, *f*_abs,CDOM_ is wavelength-dependent and is higher in the UV range than in the visible range,^53^ but most *f*_abs,CDOM_ values are measured for visible wavelengths. Additionally, in coastal and marine environments, CDOM absorbance, and therefore it's fractional importance to total absorbance, is low.^42^ For these reasons, a *f*_abs,CDOM_ value that represents an average of the available data collected within the UV range (0.7) was used, but we note that there is a large uncertainty in this value.

### Estimated [^1^O_2_]_SS_ at the near-surface of lakes

**Fig. 6:**
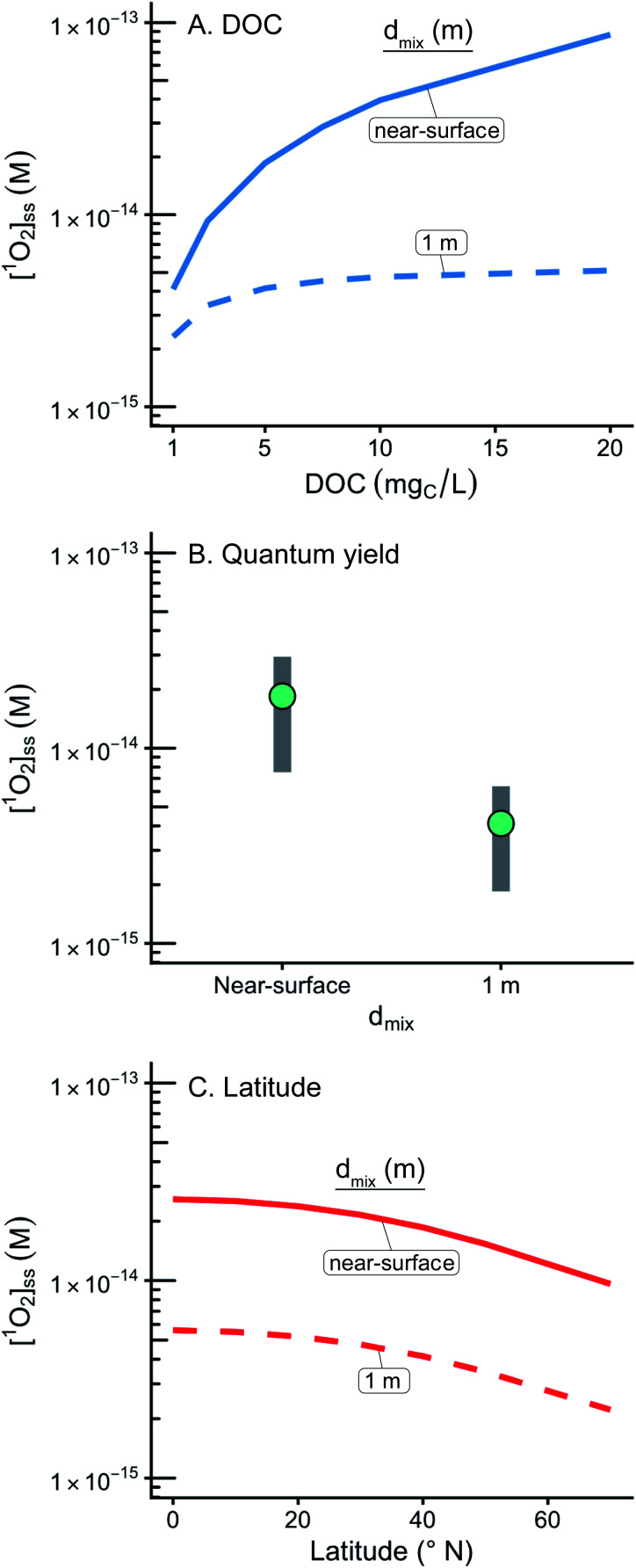
Impact of variable input parameters on [^1^O_2_]_SS_ at the near surface (defined as having a 1 cm epilimnion depth) and within a 1 m epilimnion. In panels A, B, and C, one parameter is varied, while the rest of the parameters are held constant at their “representative case” values (see Table 1). (A) Range of possible [^1^O_2_]_SS_ values for DOC concentrations ranging from 1 to 20 mg_C_ L^−1^. (B) Range of possible [^1^O_2_]_SS_ values for a range of modelled *Φ*_Δ_ relationships. Green points represent the average case. (C) Range of possible [^1^O_2_]_SS_ values for latitudes ranging from the equator to 70°N.

While predictions of [^1^O_2_]^epi,avg^_SS_ within the epilimnia of lakes are broadly applicable to modelling pollutant fate in the environment, the behavior of [^1^O_2_]_SS_ at the near-surface of a water body is sufficiently different to warrant a separate discussion. Specifically, the near-surface of a water body (defined here as having a 1 cm epilimnion) and shallow water bodies (defined here as having a 1 m epilimnion) have particular relevance in engineered environments such as waste stabilization ponds, constructed wetlands, and mesocosm experiments.^67–69^ In addition, the near-surface condition is relevant in pollutant fate and transport regulations^54^ and also represents the bulk of the literature values for [^1^O_2_]_SS_. Near-surface literature values for [^1^O_2_]_SS_ range from 4.8 × 10^−15^ to 5.6 × 10^−12^ M and vary as a function of water source and light source (Fig. S12 and Table S4†).^13,18,21,70–79^ This range of literature values is in agreement with the ranges of estimated [^1^O_2_]_SS_ values at the near-surface and in shallow water bodies (Fig. 6 and Table 4) and with the estimated near-surface [^1^O_2_]_SS_ value of 1.9 × 10^−14^ M obtained for the representative case (5.0 mg_C_ L^−1^, 40° N, average *Φ*_Δ_ as a function of wavelength relationship).

**Table 4:** Impact of different parameters on the variability of [^1^O_2_]_SS_ at the near surface (1 cm mixed layer). One parameter is varied at a time, while the rest are representative values (5.0 mg_C_ L^−1^, 40° N, average *Φ*_Δ_ as a function of wavelength relationship)

Parameter being varied	Range of values (test set)	[^1^O_2_]_SS_ (×10^−14^ M)	Factor difference between max and min values
DOC (mg_C_ L^−1^)	1.0	0.41	21
	20.0	8.7	
QY (%)	Lower limit^a^	0.75	3.9
	Upper limit^a^	2.9	
Latitude (° N)	0	2.6	2.7
	70	0.96	

a see Fig. 1A.

At the near-surface, calculated [^1^O_2_]_SS_ concentrations are one to two orders of magnitude higher than those calculated within an average (9 m) epilimnion. These significantly higher [^1^O_2_]_SS_ values occur because the incident irradiance has not been attenuated nor has the ^1^O_2_ formed at the surface been diluted within the epilimnion. The difference in [^1^O_2_]_SS_ values also manifests in the differing importance of the DOC concentration. For epilimnion depths of 9 m, changing DOC concentrations from 1–20 mg_C_ L^−1^ results in a 14% difference in [^1^O_2_]^epi,avg^_SS_ whereas at the near-surface the same change in DOC concentration results in an increase in [^1^O_2_]_SS_ by over a factor of 20.

### Estimated pollutant half-lives

**Fig. 7:**
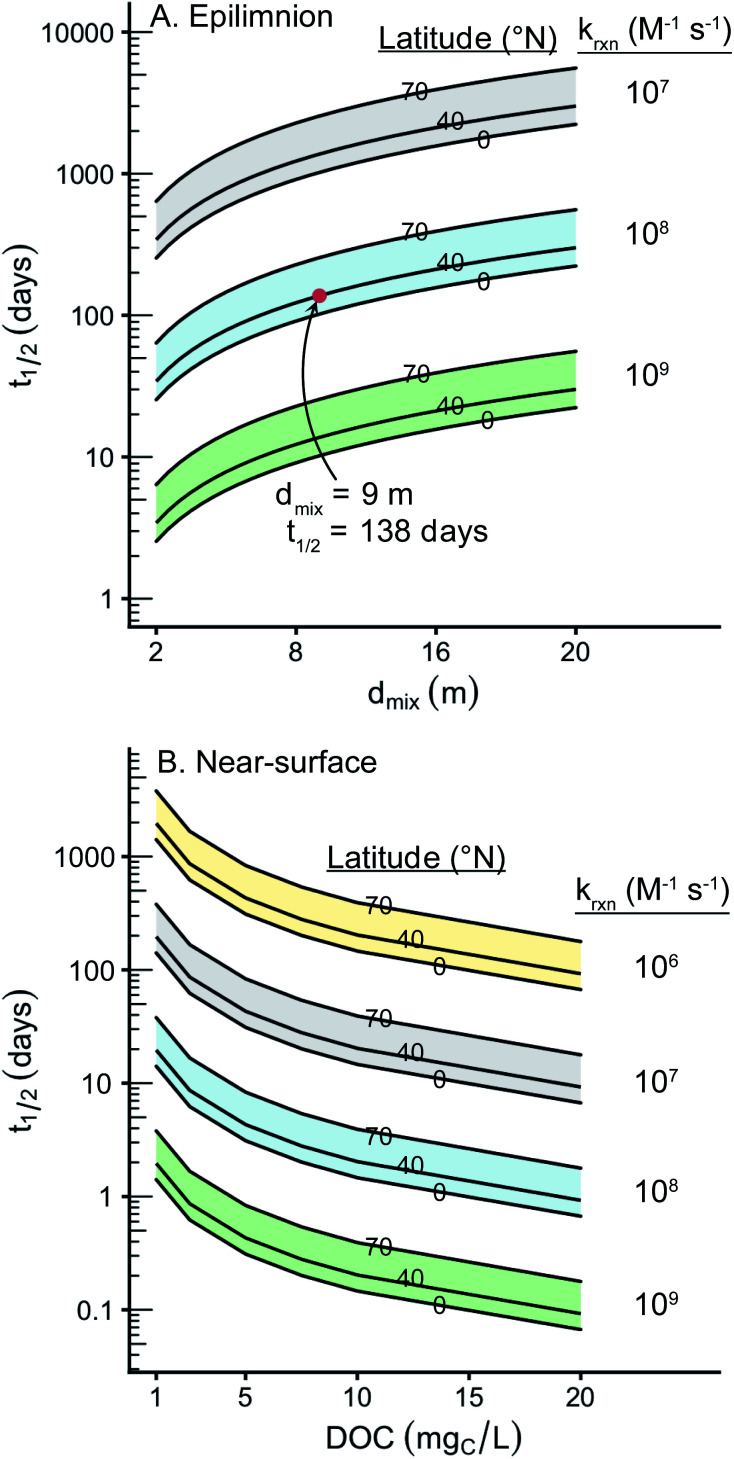
(A) Pollutant half-life in the epilimnion as a function of epilimnion depth for a DOC concentration of 5 mg_C_ L^−1^. The red point represents the half-life of an average compound in a representative water. (B) Pollutant half-life as a function of DOC concentration at the near-surface. For both plots, each shaded group of curves represents a different reaction rate constant of a hypothetical compound with ^1^O_2_ over latitudes ranging from 0 degrees to 70 degrees N.

Pollutant half-lives within the epilimnia and at the near-surface of lakes were calculated using the estimated values of [^1^O_2_]_SS_ and bimolecular rate constants of 10^6^ to 10^9^ M^−1^ s^−1^ (Fig. 7). This analysis shows that for lakes with epilimnia between 2 and 20 m the bimolecular rate constant of the pollutant of interest defines the timescale of the pollutant's half-life (Table S7†). For compounds that have very slow bimolecular rate constants such as sulfamethoxazole (2 (±1) × 10^4^ M^−1^ s^−1^),^80^ reaction with ^1^O_2_ will also be very slow, even at the near-surface (*i.e.*, *t*_1/2_ ≈ 40 years). For compounds with bimolecular rate constants on the order of 10^7^ M^−1^ s^−1^, reaction with singlet oxygen leads to half-lives on the order of years for most lakes with epilimnia greater than 2 m.

For compounds that react quickly with ^1^O_2_, such as cimetidine (2.2 (±0.2) × 10^8^ M^−1^ s^−1^ at a pH of 8.2),^6^ reaction with ^1^O_2_ results in half-lives of less than a week at the near-surface of a representative lake. When considering a representative lake with an average epilimnion depth (*d*_mix_ = 9 m), the half-life is approximately 4.5 months. In lakes with an epilimnion deeper than 9 m or at more polar latitudes, the predicted half-life can be a year or more.

### Limitations and applications of the estimates presented here

It is important to note that we have not focused on estimating the [^1^O_2_]^epi,avg^_SS_ in any one particular lake. Rather, by using ranges of values for the input parameters that impact [^1^O_2_]^epi,avg^_SS_, a deeper understanding of the [^1^O_2_]^epi,avg^_SS_ values that are possible within the epilimnia of a variety of lakes is presented. This approach requires some simplifying assumptions. Specifically, lakes have been treated as a simplified box model. This means that changes in mixed depth and [^1^O_2_]^epi,avg^_SS_ due to seasonal lake turnover or due to seasonal changes such as ice cover are not incorporated. As the focus of this work is on improving the estimate of environmentally representative values for [^1^O_2_]^epi,avg^_SS_ rather than on modelling pollutant dynamics, the half-life estimates presented here are most relevant for pollutants whose residence time within a lake is on the order of days to months. These half-lives offer an estimate of how pollutant lifetimes are impacted by factors such as epilimnion depth, latitude, singlet oxygen quantum yield, and pollutant bimolecular rate constant.

Some of the parameters considered in this work, such as *K*_d_ and *f*_a,CDOM_, are likely to co-vary, and interactions between such parameters have not explicitly been accounted for in this work. Similarly, not all combinations of parameters are equally probable. This would lead to either a narrower range of predicted [^1^O_2_]_SS_ values or different regimes of values depending on sets of parameters valid for a specific environment or lake type. By not excluding any combination of parameters, we provide a wide range of possible values of [^1^O_2_]_SS_ in this work, which we expect to narrow as future work provides better values for the parameters.

In previous sections additional limitations regarding other simplifications in our system have been noted, including the difficulty in estimating the impacts of cloud cover on the irradiance incident to a lake, the broad range of possible wavelength-dependent *Φ*_Δ_ values for different types of DOM, and the lack of generalizable data for the fraction of light attenuation due to absorbance by CDOM. The impact of including many of these factors would be to decrease the [^1^O_2_]^epi,avg^_SS_, though by how much is currently unknown.

The equations used here could be adapted to a specific lake if experimental data on the *Φ*_Δ_ and absorption coefficient of the DOM in that lake as well as information on lake dynamics were known. The framework presented here provides a basis for more environmentally representative values of [^1^O_2_]^epi,avg^_SS_ that can be used by researchers and practitioners to estimate indirect photodegradation rates in lakes.

A similar approach could in principle be used to estimate the concentrations of other PPRIs, such as hydroxyl radicals (OH˙) or triplet excited state CDOM, in sunlit lakes. However, the formation and quenching processes for other PPRIs are more complicated than those of ^1^O_2_ and in many cases are less well understood. To take OH˙ as an example, while there is evidence that CDOM is the main source of OH˙ in surface waters, other sources include nitrate and nitrite.^81,82^ The main scavenger of OH˙ in surface waters is CDOM, but in seawater bromide is the most important scavenger, while in some freshwaters carbonate and bicarbonate might also be relevant sinks.^81,83^ For each of these pathways the range of concentrations of these constituents in surface waters must be estimated, as well as the quantum yield of formation of OH˙ from the relevant sources. The more complicated dynamics of OH˙ formation and quenching increase the uncertainty of the resulting steady-state concentration such that the range of values obtained is not useful for predictions of pollutant half-lives. That being said, [OH˙]_SS_ is generally known to be around two orders of magnitude lower than [^1^O_2_]_SS_ in sunlit surface waters. Thus, as a first approximation, one could estimate depth-averaged values of [OH˙]_SS_ to be 6 × 10^−19^ to 5 × 10^−17^ M, similar to previous depth-averaged estimates.^84,85^

## Conclusions

In this work the variability of singlet oxygen steady-state concentrations within the epilimnia as well as at the near-surface of lakes has been investigated. The depth of the epilimnion is the environmental factor that most impacts the mixed-layer [^1^O_2_]^epi,avg^_SS_, while DOC concentration has a very limited effect. This contrasts the situation at the near-surface, where the DOC concentration is a dominant factor affecting [^1^O_2_]_SS_.

Near-surface [^1^O_2_]_SS_ values and epilimnion-averaged [^1^O_2_]^epi,avg^_SS_ values differ by approximately two orders of magnitude. For estimating environmental half-lives of ^1^O_2_-reactive compounds in sunlit lakes, we suggest using a range of 6 × 10^−17^ to 5 × 10^−15^ M for depth-averaged values of [^1^O_2_]_SS_. We believe that this range of [^1^O_2_]^epi,avg^_SS_ values provides a more complete picture of ^1^O_2_ in the mixed layer of surface waters, and using these values will provide more realistic estimates of environmental half-lives of pollutants in surface waters. Our results show that for the half-life of a pollutant reacting with ^1^O_2_ to be less than one year in a representative lake, the pollutant must have a bimolecular rate constant of at least 10^8^ M^−1^ s^−1^.

This work also highlights the need for more data on parameters such as wavelength-dependent *Φ*_Δ_ relationships and fraction of absorbance in surface waters that is due to CDOM. Variability in both of these parameters leads to a large variation in possible [^1^O_2_]^epi,avg^_SS_ values. In the case of *Φ*_Δ_, though there are many single values available in the literature, their use in calculations of 
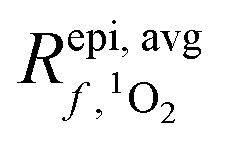
 leads to overestimations in [^1^O_2_]^epi,avg^_SS_. For *f*_abs,CDOM_, many of the available literature values are for visible wavelengths and marine environments, limiting their applicability to photochemical generation of ^1^O_2_ in surface waters. A more complete understanding of the distribution of these two parameters within surface waters globally would lead to less variability and more confident predictions of [^1^O_2_]^epi,avg^_SS_ within the epilimnia of lakes.

Finally, this work has implications for practitioners and researchers designing engineered systems that rely on photodegradation as a treatment mechanism. Our work shows that once all the photons entering a system have been absorbed, increasing the depth leads to lower [^1^O_2_]^epi,avg^_SS_ values. Similarly, for shallow ponds (*i.e.* ≤1 m deep), increasing the DOC concentration can lead to large increases in [^1^O_2_]_SS_. The data provided in Tables 2 and S5† can help researchers optimize engineered systems to maximize the production of ^1^O_2_ within the system.

## Conflicts of interest

There are no conflicts of interest to declare.

## Supplementary Material

EM-023-D1EM00062D-s001
