# Supplementary material for: Factors affecting the mixed-layer concentrations of singlet oxygen in sunlit lakes
Source: Environ Sci Process Impacts. 2021 Jun 23;23(8):1130–45. doi: 10.1039/d1em00062d (PMC8372756; doi:10.1039/d1em00062d)
Supplement: EM-023-D1EM00062D-s001 [file EM-023-D1EM00062D-s001.pdf]

Electronic Supplementary Material (ESI) for Environmental Science: Processes & Impacts.
This journal is © The Royal Society of Chemistry 2021

Supporting Information

Factors affecting the mixed-layer concentrations of singlet oxygen in sunlit lakes

Sarah B. Partanen[†], Jennifer N. Apell*[†‡], Jianming Lin[§] and Kristopher McNeill*[†]

[†]*Institute of Biogeochemistry and Pollutant Dynamics (IBP), Department of Environmental Systems Science, ETH Zurich, 8092 Zurich, Switzerland*

[§] *Firmenich Incorporated, P.O.Box 5880, Princeton, New Jersey 08543, U.S.A.*

[‡] *Department of Civil and Urban Engineering, New York University Tandon School of Engineering, 6 MetroTech Center, Brooklyn, NY 11201*

Pages: 24

Figures: 15

Tables: 7

Epilimnion depth and lake depth

Global Lakes Database

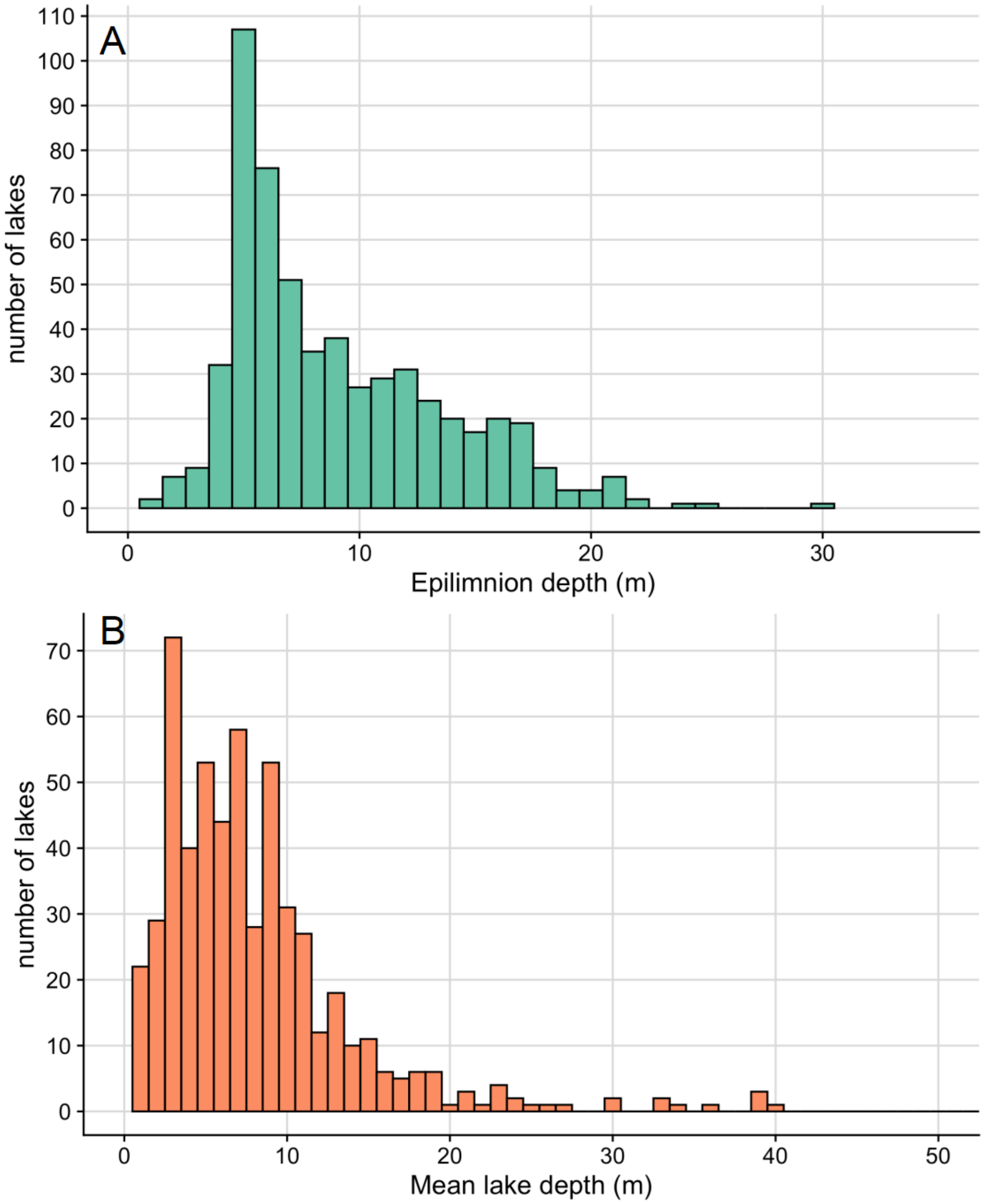

Figure S1. (A) Histogram plot of the epilimnion depth of the lakes in the Global Lakes database.[1] (B) Histogram plot of the mean depth of the lakes in the Global Lakes database. In both panels the bin size = 5m.

Table S1. Statistics describing the lakes in the Global Lakes database[1]

Parameter	Value
Number of lakes in database	573
% of lakes that are fully mixed	13
% of lakes with an epilimnion between 2 and 20 m	96
Mean epilimnion depth (m)	9.1
Median epilimnion depth (m)	7.6
Most frequently appearing epilimnion depth (m) (n = 16)	4.6

Difference between epilimnion depth and lake depth

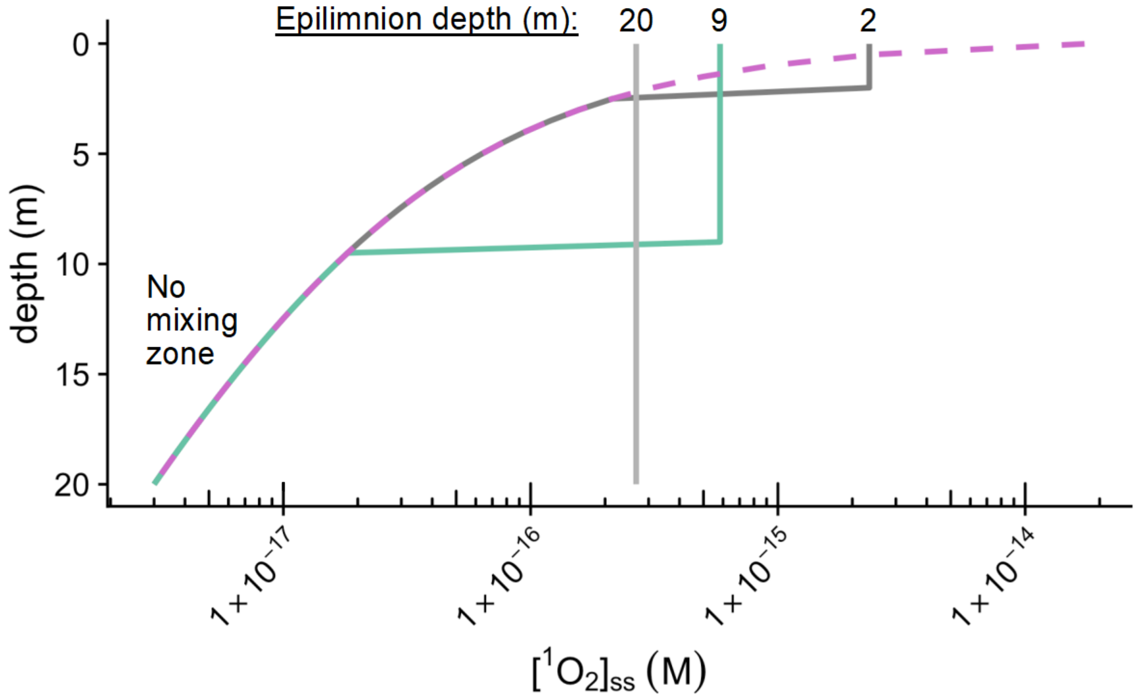

Figure S2. $[^1O_2]_{SS}$ as a function of depth (dotted purple line) and three different epilimnion depths. All other parameters are set at their representative values (see Table 1 in the main text). Note that the $[^1O_2]_{SS}$ is a constant value over the depth of the epilimnion, and below the epilimnion varies as a function of water depth.

Quantum yield relationships

$\Phi_{\Delta,\lambda}$ relationships were modelled by fitting experimental data from Partanen et al.[2] to a bi-exponential model, shown in equation S1)

$$\Phi_{\Delta,\lambda}^{predicted} = a_1 \exp(-b_1\lambda) + a_2 \exp(-b_2\lambda) \qquad \text{(S1)}$$

Where $\Phi_{\Delta,\lambda}^{predicted}$ is the modelled singlet oxygen quantum yield at a given wavelength (λ, nm), and a_1, a_2, b_1, and b_2 are fitting parameters.

The upper and lower bounds of the range of $\Phi_{\Delta,\lambda}$ values used in this work were obtained by adjusting the fitting parameters so that the experimental data was enclosed within the bounds of the modelled curves. The representative $\Phi_{\Delta,\lambda}$ curve was taken as the average of these upper and lower bounds. Figure S3 shows the experimental data overlaid on the modelled range of $\Phi_{\Delta,\lambda}$ values used in this work.

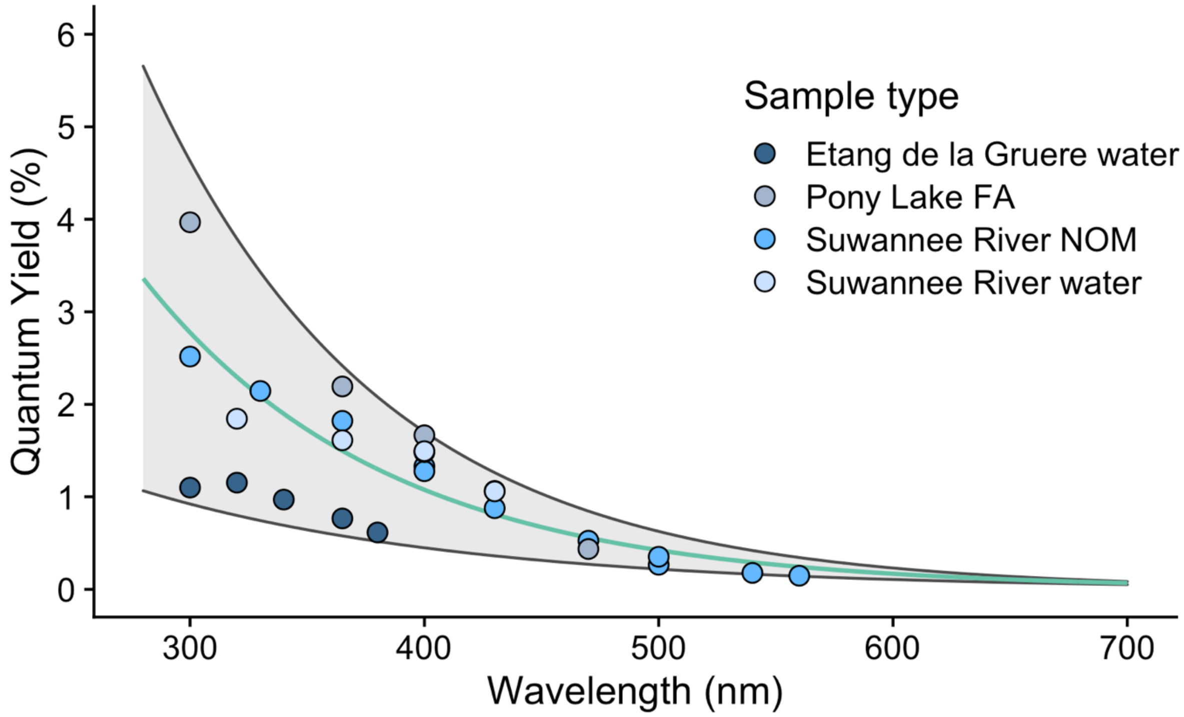

Figure S3 Modelled range of $\Phi_{\Delta,\lambda}$ values used in this work (as presented in the main text Figure 1A) along with points representing experimental $\Phi_{\Delta,\lambda}$ data measured in Partanen et al.[2]

Biexponential fits of SRNOM and PLFA

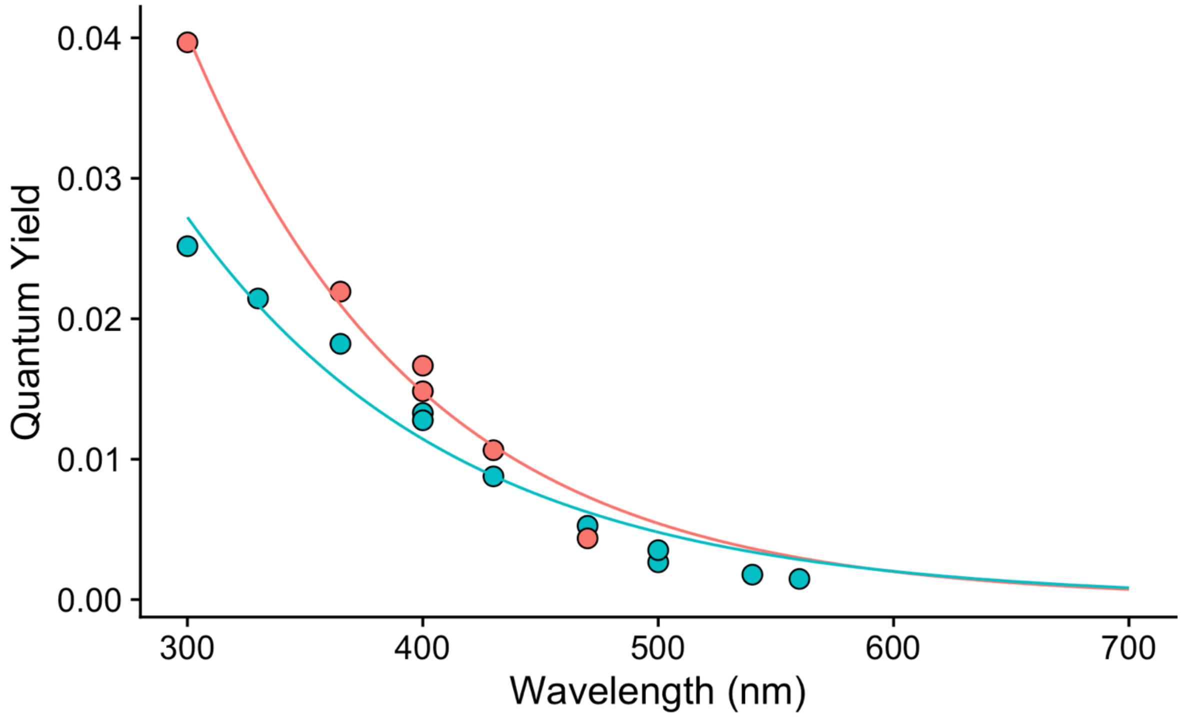

Figure S4. Biexponential fits to experimental wavelength-dependent Φ_Δ data. PLFA is shown in red, and SRNOM is shown in blue.

Factors affecting incident irradiance

Impact of reflectance on $[{}^1O_2]_{SS}^{epi,avg}$

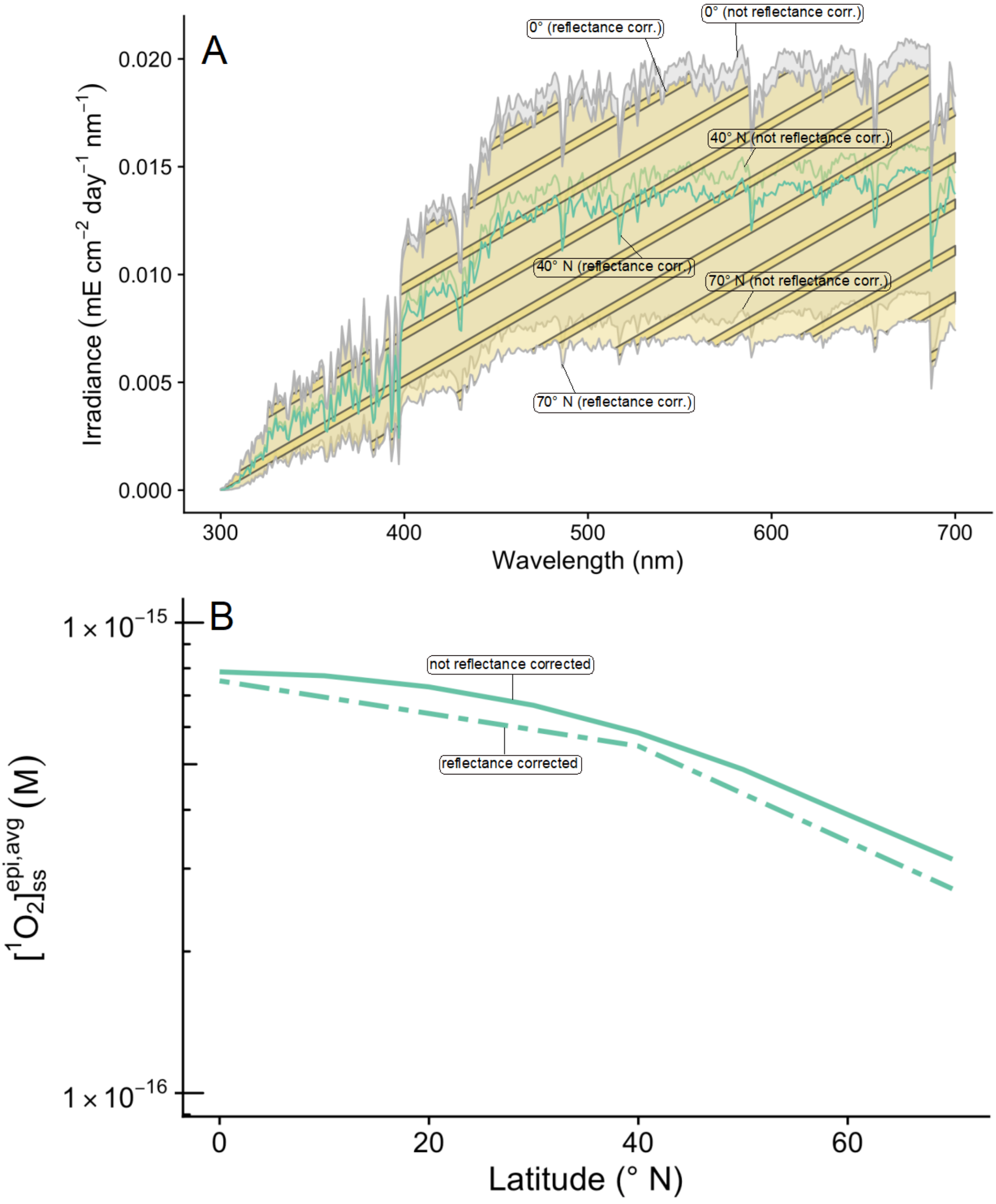

Figure S5. (A) Range of solar irradiance spectra highlighting 40°N in green. Reflectance-corrected spectra shown in patterned yellow, spectra that have not been reflectance-corrected are shown in grey for comparison. (B) Relationship between latitude and $[{}^1O_2]_{SS}$ for a 9 m epilimnion depth. $[{}^1O_2]_{SS}$ values modeled using reflectance-corrected solar irradiance spectra are shown as a dashed line.

Table S2. Percent difference between incident irradiance spectra and reflectance-corrected incident irradiance spectra for UVA, UVB and PAR wavelengths, as well as the entire solar spectrum (280 – 800 nm).

Latitude	% diff. between non-adjusted irradiance and reflectance adjusted irradiance			
	**UVB**	**UVA**	**PAR**	**Solar spectrum**
0	3.3	4.3	4.5	4.5
40	4.3	6.4	6.6	6.7
70	8.2	14	15	15

Reflectance calculations

To calculate the fraction of light transmitted into a water body, Fresnel's equations were used (eq. S2-S4).

$$t = 1 - \frac{r_s + r_p}{2} \quad \text{(S2)}$$

$$r_s = \left(\frac{\sin(\theta_i - \theta_t)}{\sin(\theta_i + \theta_t)}\right)^2 \quad \text{(S3)}$$

$$r_p = \left(\frac{\tan(\theta_i - \theta_t)}{\tan(\theta_i + \theta_t)}\right)^2 \quad \text{(S4)}$$

Where t is the fraction of light transmitted, r_s is the fraction of s-polarized light reflected, r_p is the fraction of p-polarized light reflected, θ_i is the angle of incident light, and θ_t is the angle of transmitted light.

θ_t was calculated using Snell's Law:

$$\frac{\eta_{water}}{\eta_{air}} = \frac{sin\theta_i}{sin\theta_t} \quad \text{(S5)}$$

where η_{water} is the index of refraction of water, and η_{air} is the index of refraction in air. η_{air} is 1.0003 over the UV and visible spectra, but η_{water} varies as a function of wavelength. The empirical relationship in equation S6 was used to calculate η_{water}[3]

$$\eta_{water} = 1.31405 + \frac{15.868}{\lambda} - \frac{4382}{\lambda^2} + \frac{1.1455\times10^6}{\lambda^3} \quad \text{(S6)}$$

The angle of incident light, or zenith angle, is calculated according to equation S7:

$$cos\theta_i = \sin(lat.)\sin(\delta) + \cos(lat.)\cos(h) \quad \text{(S7)}$$

where *lat.* is the latitude (° N or S), δ is the solar declination angle, and h is the hour angle.

h is calculated according to equation S8:

$$h = 15° \times (local\ solar\ time - 12) \quad \text{(S8)}$$

where local solar time are hours of the day (i.e. 0, 1, 2… 21, 22, 23).

The solar declination angle, δ, varies as a function of time of year, as seen in table S3.

Table S3. Solar declination angles for the solstices and equinoxes

Day	solar declination angle (°)
December 21	-23.44
March 21	0
June 21	23.44
September 21	0

Impact of using solar noon or summer day irradiance spectra on $[^1O_2]_{SS}^{epi,avg}$

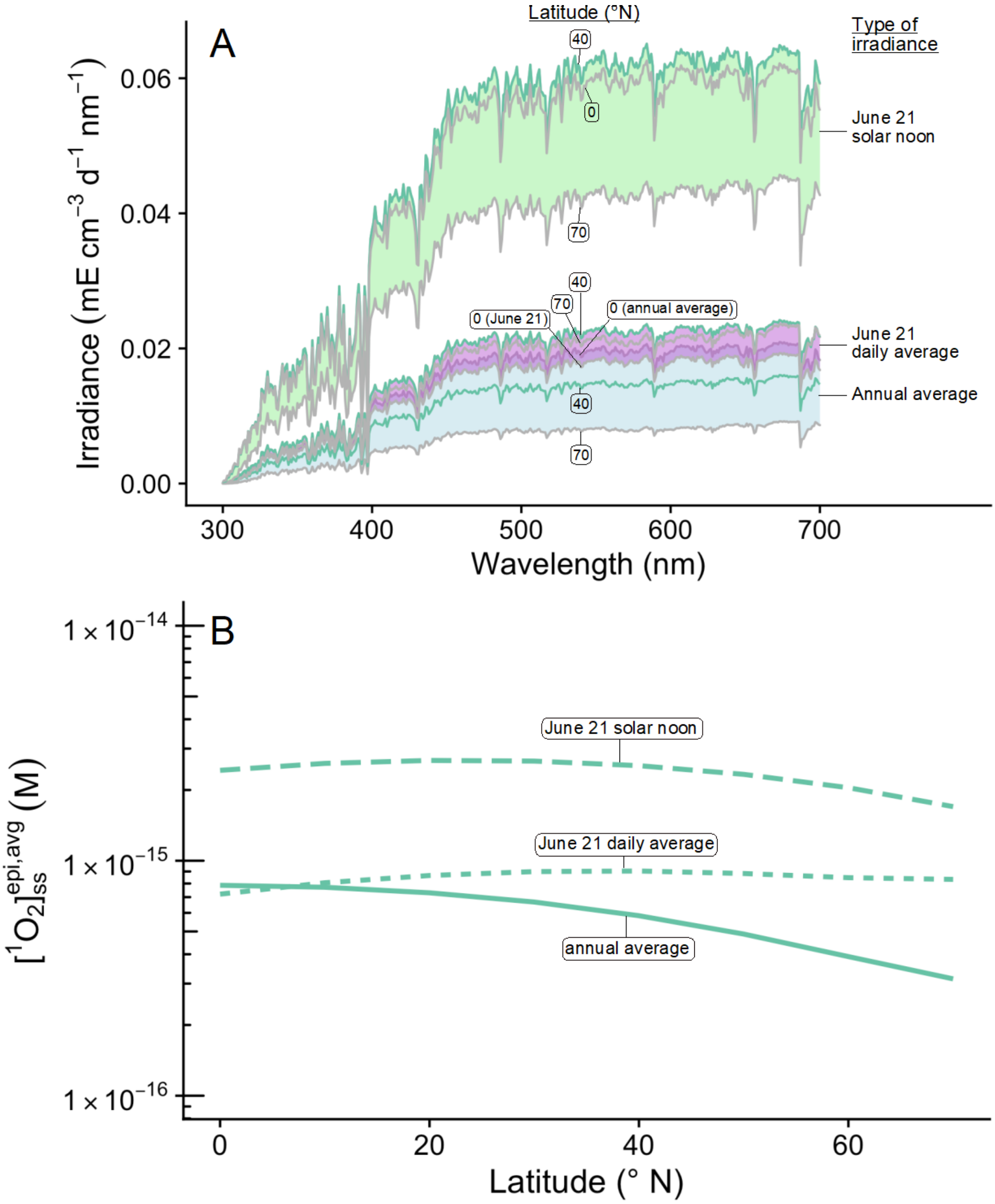

Figure S6. (A) The range of solar irradiance spectra for June 21 solar noon (green), June 21 daily average (purple), and annual average (blue). 40° N is shown as a geen line for each type of spectra. (B) Impact of using June 21 solar noon and June 21 daily average irradiance instead of average annual irradiance on calculations of $[^1O_2]_{SS}$ at a 9 m epilimnion depth.

Diffuse attenuation coefficients and absorbance

The impact of DOC concentration on $[^1O_2]_{SS}$

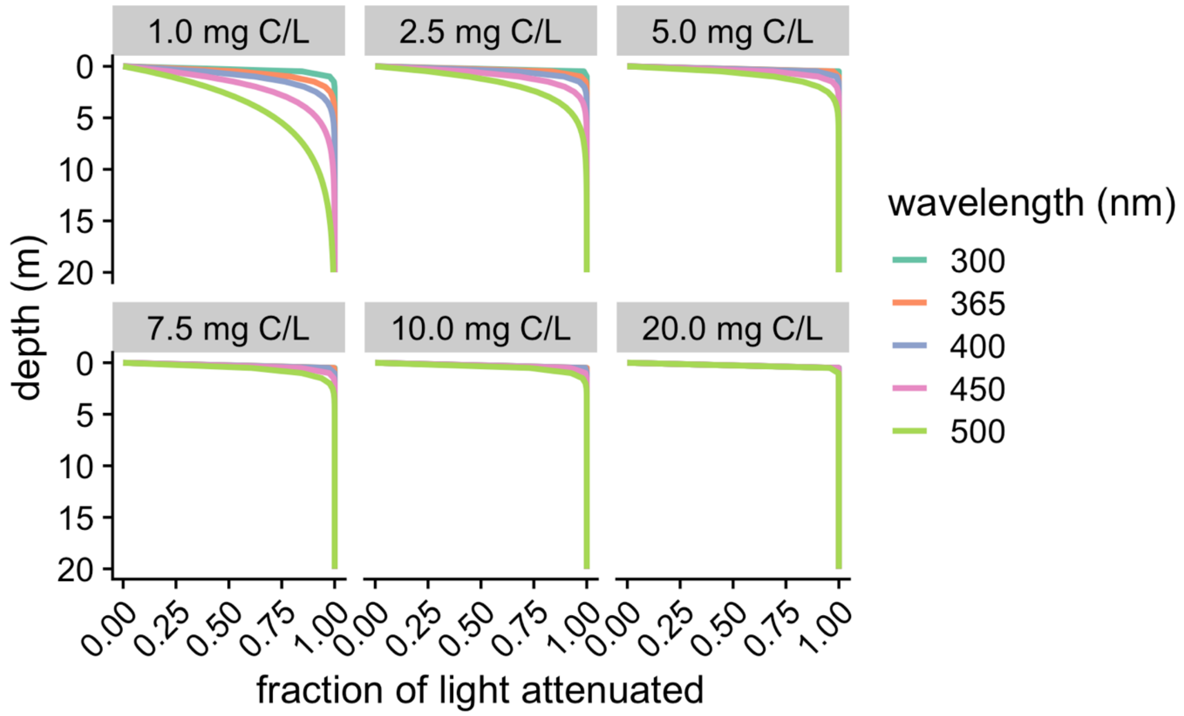

Figure S7. The modelled fraction of light attenuated as a function of water column depth for multiple DOC concentrations and wavelengths of light. These values were calcuated using an empirical relationship for K_d that takes DOC concentrations as the input variable.[4]

Equation for R_f using absorbance spectra

Since absorbance is measured in decadic log units, it is important to use the correct form of the main text equation 2. The equation shown below should be used when decadic absorbance is the input parameter rather than diffuse attenuation coefficients.

$$R_{f,{}^1O_2}^{epi,avg} = \sum_\lambda \frac{I_{0,\lambda}}{\ell} \times (1 - 10^{-\alpha_\lambda \cdot \ell}) \times \Phi_{\Delta,\lambda} \qquad \text{(S9)}$$

where α_λ is the decadic absorbance of the water (cm^{-1}), and the other parameters are as defined in the main text.

K_d vs. absorbance in calculations of $[^1O_2]_{SS}$

To compare the impact of using modeled K_d values and measured absorbance spectra on calculated values of $[^1O_2]_{SS}$, measured absorbance spectra for surface waters and organic matter isolates containing a range of DOC concentrations were used (Figure S8). Some of the surface waters were also used in the test tube validation (see below). The rest of the waters used in this analysis were collected from lakes and streams across northern and western Switzerland on July 2nd and 25th, 2019. These samples

were collected approximately 5 – 10 cm below the surface of the lake or stream and stored on ice until they could be refrigerated. Samples were passed sequentially through two filters (glass-fiber filter, pore size 0.7 μm, followed by Omnipore PTFE, pore size 0.2 μm), and stored in HDPE bottles (acid-washed, autoclaved, and wrapped in aluminum foil) at 3 ^{o}C.

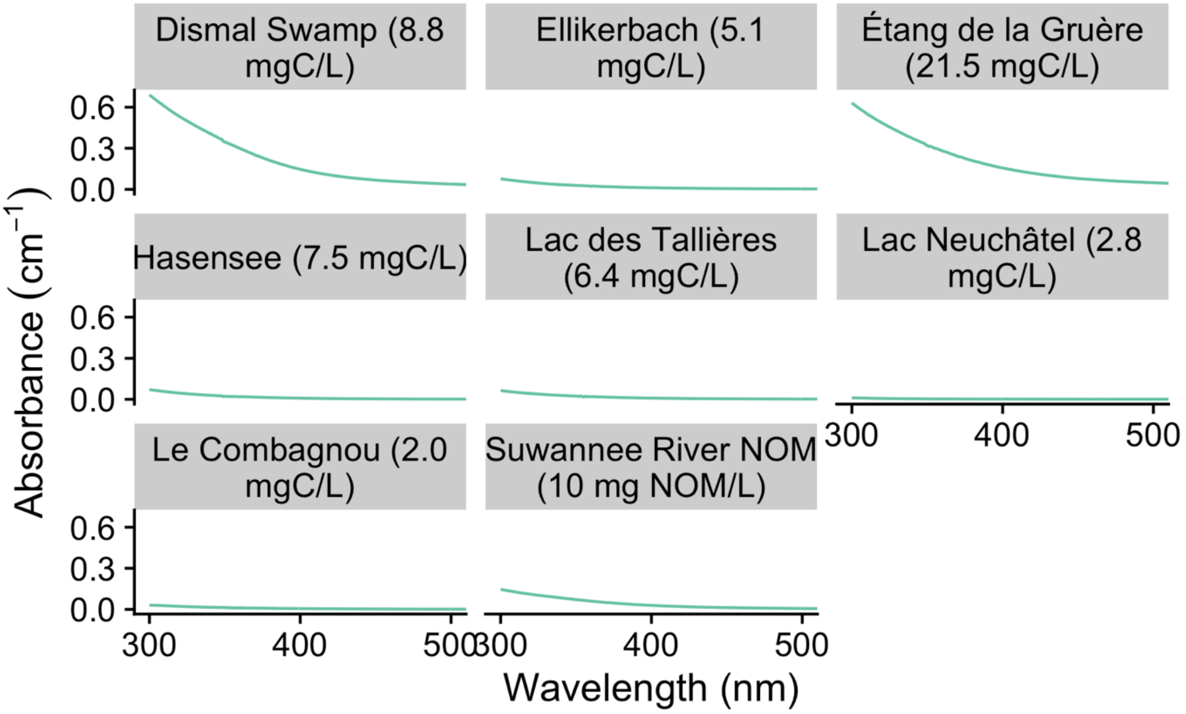

Figure S8. Absorbance spectra of the natural water samples and organic matter isolates used in a comparative analysis of the impact of experimentally measured absorbance spectra and modeled K_d values on calculations of $[^1O_2]_{SS}$.

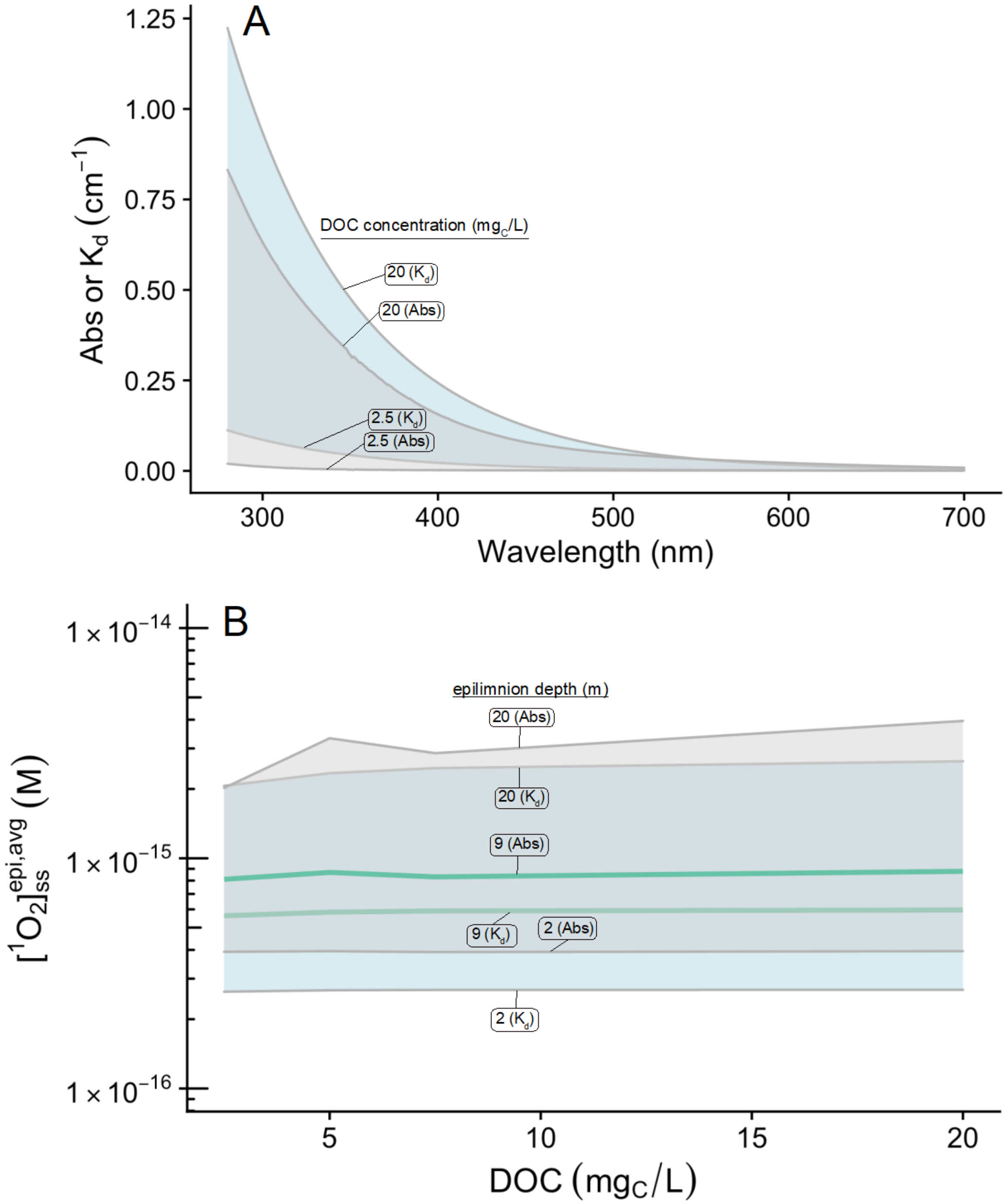

Figure S9. (A) Measured absorbance spectra for natural waters with increasing DOC concentration or diffuse attenuation coefficient spectra modelled using approximately the same DOC concentrations. (B) Relationship between DOC and $[^1O_2]_{SS}$ for a range of epilimnion depths. $[^1O_2]_{SS}$ values calculated using measured absorbance spectra are shown in grey, and those calculated using modeled diffuse attenuation coefficients are shown in blue.

Modelled absorbance spectra

It is advantageous to be able to model the absorbance spectra of surface waters, both in case it is not feasible to experimentally determine the absorbance spectra, or when a range of surface waters are of interest, not only a specific lake or river. The APEX model, for example, allows for user-input absorbance spectra as well as for approximated spectra based on the concentration of non-purgeable organic carbon (NPOC) and wavelength. This empirical relationship was developed by fitting exponential curves to experimentally obtained absorbance data for 9 lakes in northwest Italy, and is shown in equation S10 below.[5]

$$\alpha_\lambda = NPOC \cdot (0.45 \pm 0.04) \cdot e^{-(0.015 \pm 0.002)\lambda} \quad \text{(S10)}$$

$$NPOC \cong 1.3DOC \quad \text{(S11)}$$

where α_λ is the absorbance (cm^{-1}), NPOC is the concentration of non-purgeable organic carbon (mg_C/L), λ is the wavelength (nm), and DOC is the concentration of dissolved organic carbon (mg_C/L).

As seen in Figure S10, we find that absorbance spectra collected from surface waters from Switzerland and the U.S. (Figure S8) do not well match the absorbance spectra generated using the empirical relationship in equation S10. This may be because the empirical relationship was developed using waters with low NPOC concentrations (maximum NPOC = 5.4 mg_C/L, corresponding to a DOC concentration of approximately 7 mg_C/L), or it could be that the absorbance of lakes in a specific geographic location cannot be generalized to other lakes. The difficulty in generalizing absorbance spectra gives another reason to use modelled K_d values when calculating $[^1O_2]_{SS}$ in surface waters. It is also important to remember that based on the results of this work, the DOC concentration has a very limited impact on $[^1O_2]_{SS}^{epi,avg}$.

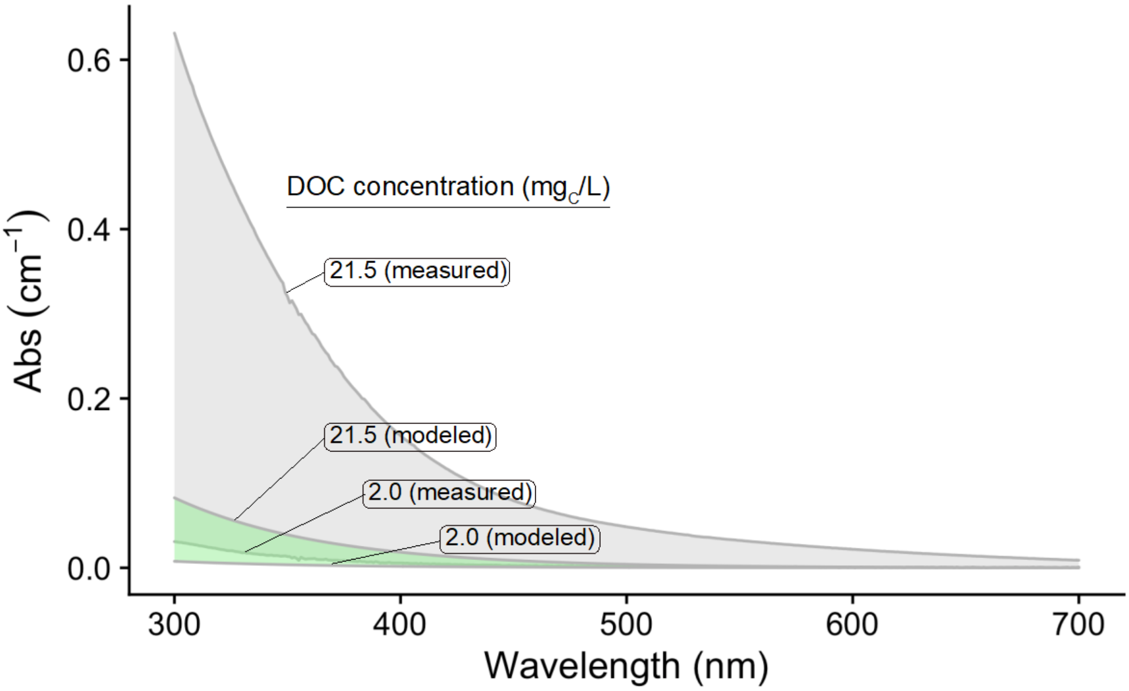

Figure S10. Absorbance modelled using an empirical relationship (green region) and measured absorbance values for corresponding DOC concentrations (grey region).

Impact of $f_{backscatter}$ and $f_{a,CDOM}$ on $[^1O_2]_{SS}^{epi,avg}$

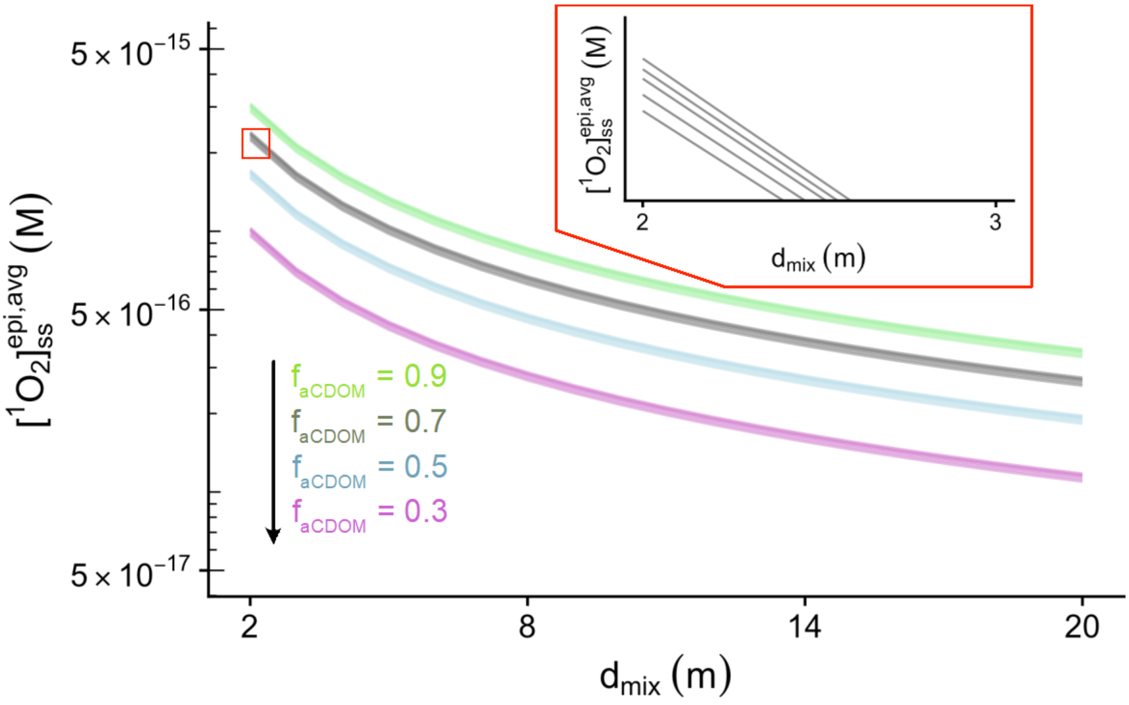

Figure S11. Impact of $f_{backscatter}$ and $f_{a,CDOM}$ on $[^1O_2]_{SS}^{epi,avg}$. The inset plot zooms in on one set of curves for $f_{a,CDOM}$ = 0.7 to better show the impact of changing $f_{backscatter}$ from 0.005 to 0.07.

$[^1O_2]_{SS}$ at the near-surface of lakes

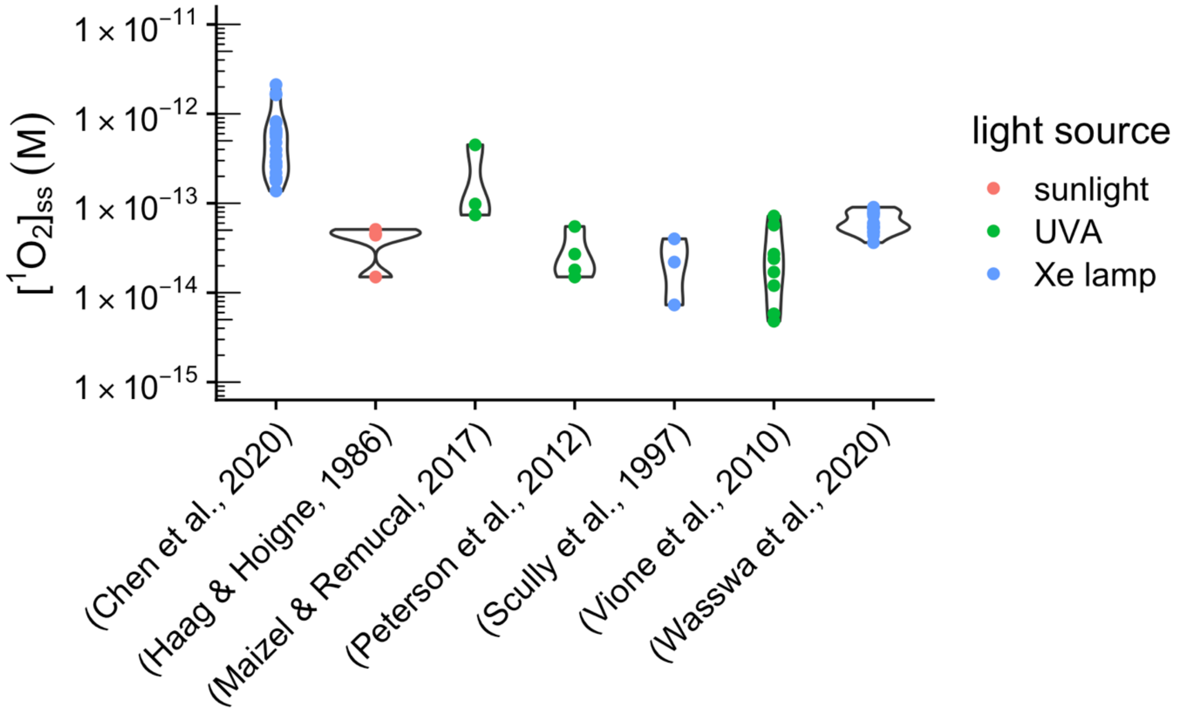

Figure S12. Range of literature $[^1O_2]_{SS}$ values for a variety of different lakes, measured under different light sources[6–9]

Table S4. Average value of $[^1O_2]_{SS}$ reported in the literature for a given water source and light source[5–16]

water source	light source	$[^1O_2]_{ss,avg}$	number of data points	reference
lake	sunlight	3.90E-14	4	(Haag & Hoigne, 1986)
lake	UVA	2.07E-13	3	(Maizel & Remucal, 2017)
lake	UVA	2.88E-14	4	(Peterson et al., 2012)
lake	UVA	2.75E-14	8	(Vione et al., 2010)
lake	Xe lamp	5.89E-13	23	(Chen et al., 2020)
lake	Xe lamp	2.31E-14	3	(Scully et al., 1997)
lake	Xe lamp	6.25E-14	16	(Wasswa et al., 2020)
river	Hg lamp	2.69E-13	1	(Li et al., 2016)
river	sunlight	3.83E-14	3	(Haag & Hoigne, 1986)
river	UVA	4.03E-12	1	(Maizel & Remucal, 2017)
river	UVA	4.45E-13	2	(Peterson et al., 2012)
river	Xe lamp	7.38E-13	1	(Chen et al., 2020)
wetland	sunlight	2.79E-13	3	(Zeng & Arnold, 2013)
wetland	UVA	3.59E-12	2	(Maizel & Remucal, 2017)
wetland	Xe lamp	1.31E-12	9	(McCabe & Arnold, 2016)
wetland	Xe lamp	7.30E-13	1	(Zeng & Arnold, 2013)
effluent	sunlight	2.77E-14	3	(Haag & Hoigne, 1986)
effluent	UVA	1.44E-12	2	(Maizel & Remucal, 2017)
effluent	Xe lamp	1.38E-13	2	(Mostafa & Rosario-Ortiz, 2013)
effluent	Xe lamp	4.14E-13	1	(Zhang et al., 2014)
effluent	Xe lamp	1.90E-13	1	(Zhou et al., 2017)

Test tube validation using natural water samples and organic matter isolates

Natural water sample collection and preservation

Natural water grab samples were obtained from Lake Bradford (Tallahassee, Florida, USA) on December 28, 2015, the Great Dismal Swamp (Virginia, USA) along the Jericho Ditch on September 8, 2016, the Suwannee River (Okefenokee Swamp, Georgia, USA) on August 9, 2017, and Étang de la Gruère (Saignelégier, Jura, Switzerland) on July 25, 2019. With the exception of Étang de la Gruère water, all samples were filtered (Whatman Polycap TC 75, pore size 0.2 μm) upon collection and stored in acid-washed brown plastic bottles at 3 °C. Étang de la Gruère water was passed sequentially through two filters (glass-fiber filter, pore size 0.7 μm, followed by Omnipore PTFE, pore size 0.2

μm) and stored in HDPE bottles (acid-washed, autoclaved, and wrapped in aluminum foil) at 3 °C.

Organic matter isolates

Suwannee River natural organic matter (SRNOM) #2R101N and Pony Lake fulvic acid (PLFA) #1R109, were purchased from the International Humic Substance Society (St. Paul, MN).

Sample characterization

Samples were characterized by measuring their absorbance spectra using a Cary 100 Biospec UV-Vis spectrophotometer, and by measuring their DOC concentration using a Shimadzu TOC-L analyzer. Φ_Δ values for each of the samples were taken from Partanen et al.[2] The DOC concentration (natural water samples) or DOM concentration (organic matter isolates) and the Φ_Δ values can be seen in Table S4, and the absorbance spectra of the samples can be seen in Figure S13.

Table S5. Characterization of surface water samples and organic matter isolates

	Concentration		
**Sample name**	**mg_C/L**	**mg DOM/L**	**Φ_Δ (%)**
Great Dismal Swamp water	8.79		1.0
Lake Bradford water	19.50		1.4
Suwannee River water	63.21		1.6
Étang de la Gruère water	21.45		0.8
Suwannee River NOM		5 and 10	1.8
Pony Lake FA		5	2.4

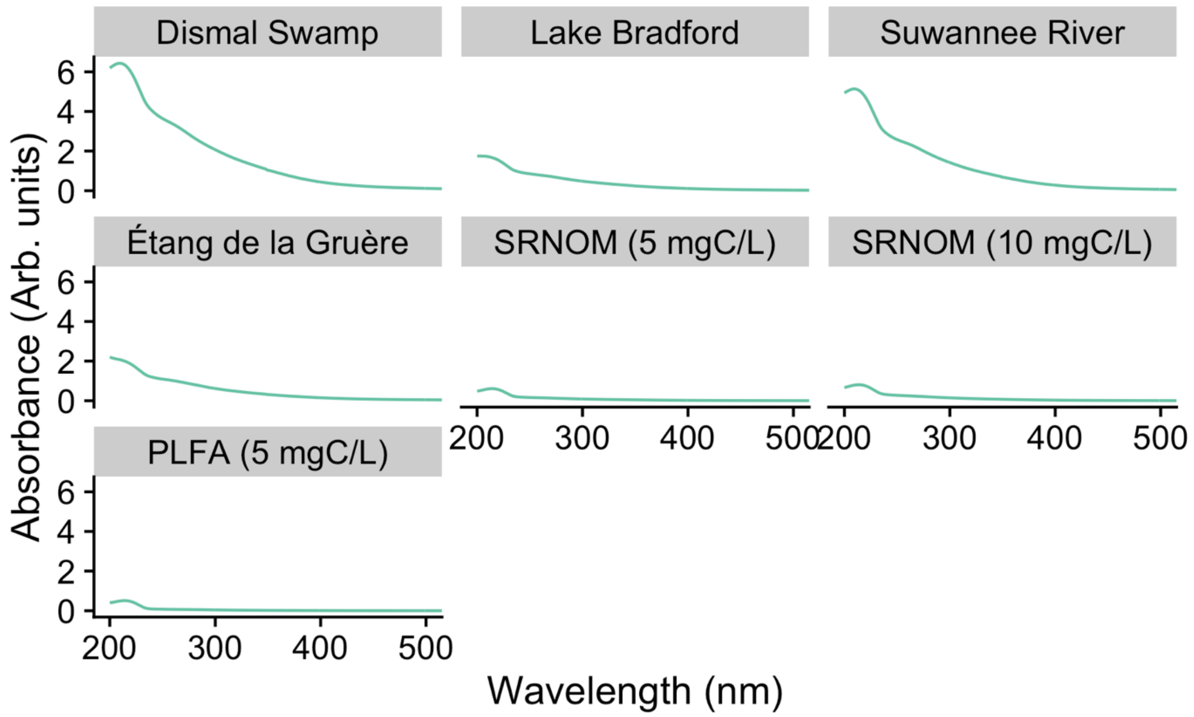

Figure S13. Absorbance spectra for surface water samples and organic matter isolates used in the test-tube validation. Note that Dismal Swamp and Suwannee River water were diluted three-fold for the absorbance measurement. The spectra presented here are corrected for this dilution.

Measurements of $[^1O_2]_{SS}$

To measure $[^1O_2]_{SS}$ of the Swiss surface water samples, steady-state irradiation experiments were performed using a Rayonet photoreactor equipped with UVA bulbs. Briefly, solutions of the surface water containing 40 µM furfuryl alcohol (FFA) as a molecular probe for $^1O_2$ were irradiated with UVA light for 90 minutes. Two different reference solutions were used; one consisted of 2 µM perinaphthenone (PN), a $^1O_2$ reference sensitizer used for calculations of Φ_Δ values (for comparison purposes only), and 40 µM FFA, and the other was a chemical actinometer containing 10 µM p-nitroanisole (PNA) used for calculating absolute irradiance values. Aliquots of the samples and references were taken periodically, and the degradation of FFA or PNA was measured using a Dionex Ultimate HPLC-UV-FLD.

$[^1O_2]_{SS}$ in the experimental solutions was calculated using the following equation:

$$k_{obs}^{FFA} = k_{rxn,^1O_2}^{FFA} \times [^1O_2]_{SS} \qquad \text{(S12)}$$

where k_{obs}^{FFA} is the observed FFA degradation rate constant in the experimental solution (s^{-1}), and $k_{rxn,^1O_2}^{FFA}$ is the bimolecular rate constant of FFA reaction with $^1O_2$. k_{obs}^{FFA} is

obtained from the slope of a plot of $\ln\left(\frac{A_{FFA}}{A_{FFA,0}}\right)$ versus time, where A_{FFA} is the area under the HPLC signal for FFA at a given timepoint, and $A_{FFA,0}$ is the area under the HPLC signal for FFA at time = 0. Plots of $\ln\left(\frac{A_{FFA}}{A_{FFA,0}}\right)$ versus time for the natural water samples, organic matter isolates, and reference sensitizers can be seen in Figure S14. $k^{FFA}_{rxn,{}^1O_2} = 1.0 \times 10^8 M^{-1}s^{-1}$ at 22°C.[17]

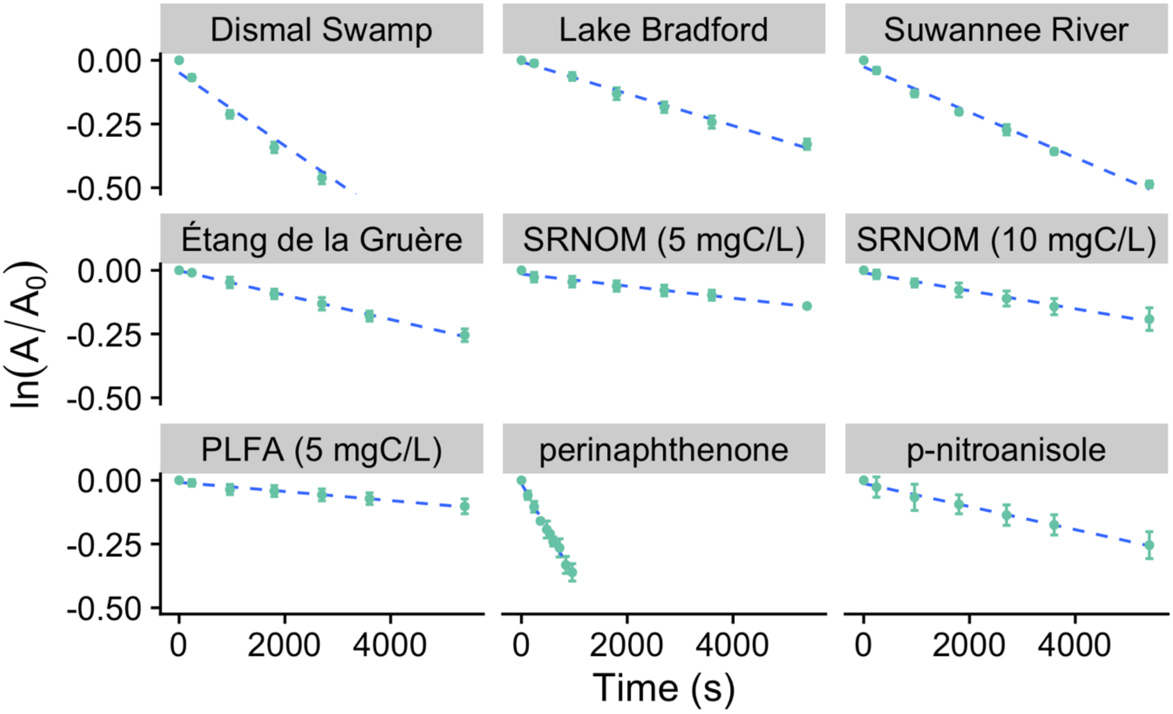

Figure S14. Plots of $\ln(A/A_0)$ vs. time for the samples tested. Results represent triplicate measurements

For calculations of $[{}^1O_2]_{SS}$ within the surface water samples and organic matter isolate solutions using equations 1 (main text) and S9, values of $I_{0,\lambda}$, sample absorbance, and Φ_Δ must be known. The measured sample absorbances shown in Figure S13 were used, as were the Φ_Δ values in Table S4, as described above. Note that these quantum yields are not wavelength dependent but are valid for the irradiance spectrum of the UVA lamp used in this validation. To calculate $I_{0,\lambda}$, the irradiance spectrum of the UVA bulbs used to irradiate the samples (Figure S15) needed to be converted from the arbitrary units obtained by an uncalibrated radiometer (Ocean Optics Jaz Radiometer) to absolute irradiance (mmol photons cm^{-2} s^{-1} nm^{-1}) using a chemical actinometer that was irradiated alongside the surface water samples. The following equations were used:

$$I_{0,\lambda} = \gamma \cdot I_{\lambda,rel} \quad \text{(S13)}$$

where γ is a wavelength-independent scaling factor, and $I_{\lambda,rel}$ is the relative irradiance (unitless).

The relative irradiance is calculated according to equation S14:[18]

$$I_{\lambda,rel} = \frac{I_{\lambda,m}}{\sum_{\lambda} I_{\lambda,m}} \quad \text{(S14)}$$

where $I_{\lambda,m}$ is the irradiance spectrum collected from an uncalibrated radiometer.

γ is calculated according to equation S15:

$$\gamma = \frac{k_{obs,PNA}}{2.303 \cdot \Phi_{dir}^{PNA} \sum_{\lambda} I_{\lambda,rel} \cdot \varepsilon_{\lambda}^{PNA} \cdot S_{\lambda}^{PNA} \Delta\lambda} \quad \text{(S15)}$$

where $k_{obs,PNA}$ is the observed PNA degradation rate constant (s^{-1}), Φ_{dir}^{PNA} is the direct photolysis quantum yield of PNA[18], $\varepsilon_{\lambda}^{PNA}$ are literature values for the molar extinction coefficient of PNA[18], and S_{λ}^{PNA} is the PNA solution screening factor.

The screening factor, S_{λ}^{PNA}, is defined as:

$$S_{\lambda}^{PNA} = \frac{1-10^{-\alpha_{\lambda}^{PNA}\ell}}{2.303\alpha_{\lambda}^{PNA}\ell} \quad \text{(S16)}$$

where α_{λ}^{PNA} is the absorbance of the experimental PNA solution (cm^{-1}), and ℓ is the pathlength (cm).

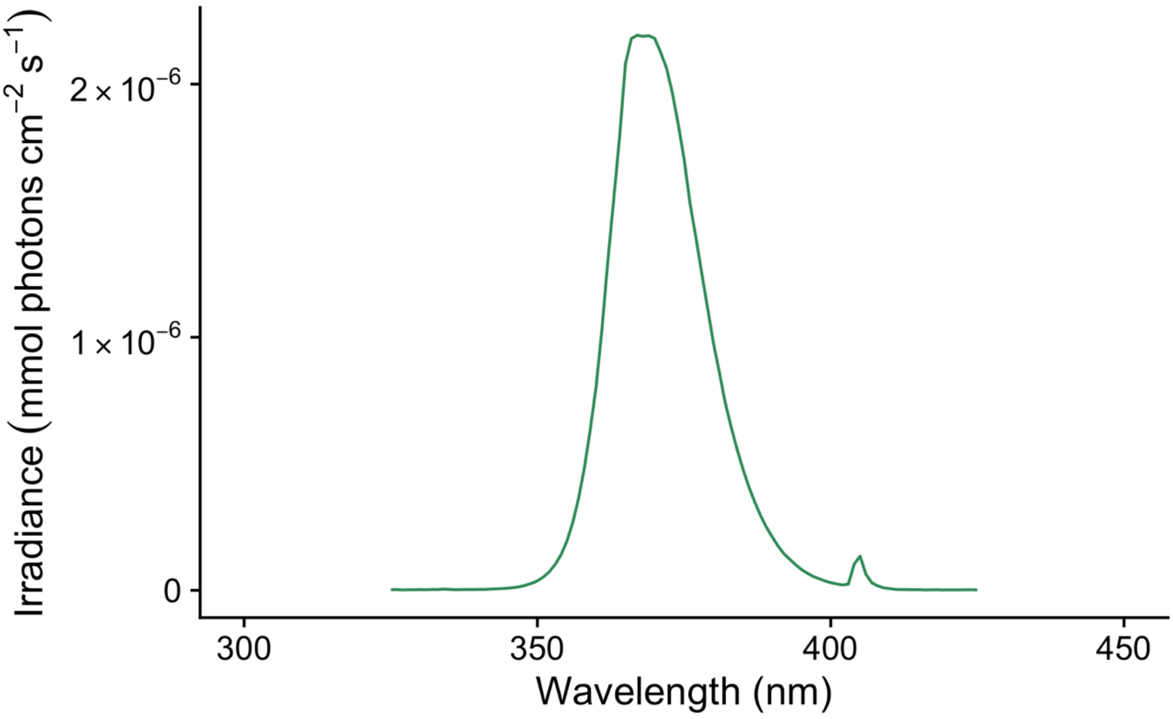

Figure S15. Absolute irradiance of the UVA bulbs used in the Rayonet photoreactor

Experimentally determined Φ_Δ values

Experimentally determined Φ_Δ values were calculated for the samples for comparison with the reference Φ_Δ values used in the analysis. Φ_Δ values were calculated using a relative method with PN as the reference sensitizer and FFA as a chemical probe for $^1O_2$ according to the following equation:

$$\Phi_\Delta^{sample} = \frac{k_{obs,FFA}^{sample}}{k_{obs,FFA}^{PN}} \times \frac{R_{abs}^{PN}}{R_{abs}^{sample}} \times \Phi_\Delta^{PN} \qquad \text{(S17)}$$

where $k_{obs,FFA}^{sample}$ and $k_{obs,FFA}^{PN}$ are the observed FFA degradation rate constants of the sample and of PN, respectively (s^{-1}), R_{abs}^{sample} and R_{abs}^{PN} are the rate of light absorbance of either the sample solution or of PN (mol photon L^{-1} s^{-1}), and Φ_Δ^{PN} is the singlet oxygen quantum yield of PN. In the following calculations we used a Φ_Δ^{PN} value of 1.01, the value measured by Schmitt et al. in pure water.[19]

Table of $[^1\boldsymbol{O_2}]_{SS}^{epi,avg}$ values for a range of parameter sets

Table S6. $[^1O_2]_{SS}^{epi,avg}$ values for a range of epilimnion depths and three parameter sets

	$[^1O_2]_{SS}{}^{epi,avg}$ (10^{-16} M)		
	Parameter sets		
**Epilimnion depth (m)**	**upper limit[a]**	**lower limit[b]**	**representative[c]**
2	54	3.7	23
3	36	2.9	16
4	27	2.4	13
5	22	2	10
6	18	1.8	8.6
7	16	1.6	7.4
8	14	1.4	6.5
9	12	1.3	5.8
10	11	1.2	5.3
11	10	1.1	4.8
12	9.2	1.0	4.4
13	8.5	0.94	4.1
14	7.8	0.88	3.8
15	7.3	0.83	3.5
16	6.9	0.78	3.3
17	6.5	0.74	3.1
18	6.1	0.71	3.0
19	5.8	0.67	2.8
20	5.5	0.64	2.7

[a] Latitude = 0°, DOC = 20 mg_C/L, upper limit Φ_Δ relationship
[b] Latitude = 70° N, DOC = 1 mg_C/L, lower limit Φ_Δ relationship
[c] Latitude = 40° N, DOC = 5 mg_C/L, representative case for Φ_Δ relationship

Range of calculated half-lives

Table S7. Range of $t_{1/2}$ values due to pollutant reaction with $^1O_2$ for a range of k_{rxn} values and epilimnion depths. These values were calculated for a water body with a DOC concentration of 5 mg_C/L at 40° N.

k_{rxn} (M^{-1} s^{-1})	Epilimnion depth (m)	$t_{1/2}$ (days)
10^9	Near-surface	0.43
	2	3.4
	9	14
	20	30
10^8	Near-surface	4.3
	2	34
	9	140
	20	300
10^7	Near-surface	43
	2	340
	9	1,380
	20	3,000
10^6	Near-surface	430
	2	3,400
	9	14,000
	20	30,000

References

(1) Qin, B.; Zhou, J.; Elser, J. J.; Gardner, W. S.; Deng, J.; Brookes, J. D. Water Depth Underpins the Relative Roles and Fates of Nitrogen and Phosphorus in Lakes. *Environ. Sci. Technol.* **2020**, *54* (6), 3191–3198. https://doi.org/10.1021/acs.est.9b05858.

(2) Partanen, S. B.; Erickson, P. R.; Latch, D. E.; Moor, K. J.; McNeill, K. Dissolved Organic Matter Singlet Oxygen Quantum Yields: Evaluation Using Time-Resolved Singlet Oxygen Phosphorescence. *Environ. Sci. Technol.* **2020**, *54* (6), 3316–3324. https://doi.org/10.1021/acs.est.9b07246.

(3) Quan, X.; Fry, E. S. Empirical Equation for the Index of Refraction of Seawater. *Appl. Opt.* **1995**, *34* (18), 3477–3480. https://doi.org/10.1364/AO.34.003477.

(4) Morris, D. P.; Zagarese, H.; Williamson, C. E.; Balseiro, E. G.; Hargreaves, B. R.; Modenutti, B.; Moeller, R.; Queimalinos, C. The Attenuation of Solar UV Radiation in Lakes and the Role of Dissolved Organic Carbon. *Limnol. Oceanogr.* **1995**, *40* (8), 1381–1391. https://doi.org/10.4319/lo.1995.40.8.1381.

(5) Vione, D.; Das, R.; Rubertelli, F.; Maurino, V.; Minero, C.; Barbati, S.; Chiron, S. Modelling the Occurrence and Reactivity of Hydroxyl Radicals in Surface Waters:

Implications for the Fate of Selected Pesticides. *Int. J. Environ. Anal. Chem.* **2010**, *90* (3–6), 260–275. https://doi.org/10.1080/03067310902894218.
(6) Haag, W. R.; Hoigné, J. Singlet Oxygen in Surface Waters. 3. Photochemical Formation and Steady-State Concentrations in Various Types of Waters. *Environ. Sci. Technol.* **1986**, *20* (4), 341–348. https://doi.org/10.1021/es00146a005.
(7) Chen, Y.; Hozalski, R. M.; Olmanson, L. G.; Page, B. P.; Finlay, J. C.; Brezonik, P. L.; Arnold, W. A. Prediction of Photochemically Produced Reactive Intermediates in Surface Waters via Satellite Remote Sensing. *Environ. Sci. Technol.* **2020**, *54* (11), 6671–6681. https://doi.org/10.1021/acs.est.0c00344.
(8) Scully, N. M.; Vincent, W. F.; Lean, D. R. S.; Cooper, W. J. Implications of Ozone Depletion for Surface-Water Photochemistry: Sensitivity of Clear Lakes. *Aquat. Sci.* **1997**, *59* (3), 260–274. https://doi.org/10.1007/BF02523277.
(9) Wasswa, J.; Driscoll, C. T.; Zeng, T. Photochemical Characterization of Surface Waters from Lakes in the Adirondack Region of New York. *Environ. Sci. Technol.* **2020**, *54* (17), 10654–10667. https://doi.org/10.1021/acs.est.0c02811.
(10) Maizel, A. C.; Remucal, C. K. The Effect of Probe Choice and Solution Conditions on the Apparent Photoreactivity of Dissolved Organic Matter. *Environ. Sci. Process. Impacts* **2017**, *19* (8), 1040–1050. https://doi.org/10.1039/c7em00235a.
(11) Peterson, B. M.; McNally, A. M.; Cory, R. M.; Thoemke, J. D.; Cotner, J. B.; McNeill, K. Spatial and Temporal Distribution of Singlet Oxygen in Lake Superior. *Environ. Sci. Technol.* **2012**, *46* (13), 7222–7229. https://doi.org/10.1021/es301105e.
(12) Li, Y.; Qiao, X.; Zhou, C.; Zhang, Y.; Fu, Z.; Chen, J. Photochemical Transformation of Sunscreen Agent Benzophenone-3 and Its Metabolite in Surface Freshwater and Seawater. *Chemosphere* **2016**, *153*, 494–499. https://doi.org/10.1016/j.chemosphere.2016.03.080.
(13) Zeng, T.; Arnold, W. A. Pesticide Photolysis in Prairie Potholes: Probing Photosensitized Processes. *Environ. Sci. Technol.* **2013**, *47* (13), 6735–6745. https://doi.org/10.1021/es3030808.
(14) McCabe, A. J.; Arnold, W. A. Seasonal and Spatial Variabilities in the Water Chemistry of Prairie Pothole Wetlands Influence the Photoproduction of Reactive Intermediates. *Chemosphere* **2016**, *155*, 640–647. https://doi.org/10.1016/j.chemosphere.2016.04.078.
(15) Mostafa, S.; Rosario-Ortiz, F. L. Singlet Oxygen Formation from Wastewater Organic Matter. *Environ. Sci. Technol.* **2013**, *47* (15), 8179–8186. https://doi.org/10.1021/es401814s.
(16) Zhou, H.; Lian, L.; Yan, S.; Song, W. Insights into the Photo-Induced Formation of Reactive Intermediates from Effluent Organic Matter: The Role of Chemical Constituents. *Water Res.* **2017**, *112*, 120–128. https://doi.org/10.1016/j.watres.2017.01.048.
(17) Appiani, E.; Ossola, R.; Latch, D. E.; Erickson, P. R.; McNeill, K. Aqueous Singlet Oxygen Reaction Kinetics of Furfuryl Alcohol: Effect of Temperature, PH, and Salt Content. *Environ. Sci. Process. Impacts* **2017**, *19* (4), 507–516. https://doi.org/10.1039/C6EM00646A.
(18) Laszakovits, J. R.; Berg, S. M.; Anderson, B. G.; O'Brien, J. E.; Wammer, K. H.; Sharpless, C. M. P-Nitroanisole/Pyridine and p-Nitroacetophenone/Pyridine Actinometers Revisited: Quantum Yield in Comparison to Ferrioxalate. *Environ. Sci. Technol. Lett.* **2017**, *4* (1), 11–14. https://doi.org/10.1021/acs.estlett.6b00422.
(19) Schmidt, R.; Tanielian, C.; Dunsbach, R.; Wolff, C. Phenalenone, a Universal Reference Compound for the Determination of Quantum Yields of Singlet Oxygen

O2(1Δg) Sensitization. *J. Photochem. Photobiol. Chem.* **1994**, *79* (1), 11–17. https://doi.org/10.1016/1010-6030(93)03746-4.
